# Revisiting Inflammatory Bowel Disease: Pathology, Treatments, Challenges and Emerging Therapeutics Including Drug Leads from Natural Products

**DOI:** 10.3390/jcm9051273

**Published:** 2020-04-28

**Authors:** Karma Yeshi, Roland Ruscher, Luke Hunter, Norelle L. Daly, Alex Loukas, Phurpa Wangchuk

**Affiliations:** 1 Centre for Molecular Therapeutics, Australian Institute of Tropical Health and Medicine, James Cook University, Cairns QLD 4878, Australia; 2 School of Chemistry, University of New South Wales (UNSW), Sydney NSW 2052, Australia

**Keywords:** inflammatory bowel diseases, ulcerative colitis, Crohn’s disease, small molecule drugs, biologics, anti-inflammatory, natural products drugs

## Abstract

Inflammatory bowel disease (IBD) is a chronic and life-long disease characterized by gastrointestinal tract inflammation. It is caused by the interplay of the host’s genetic predisposition and immune responses, and various environmental factors. Despite many treatment options, there is no cure for IBD. The increasing incidence and prevalence of IBD and lack of effective long-term treatment options have resulted in a substantial economic burden to the healthcare system worldwide. Biologics targeting inflammatory cytokines initiated a shift from symptomatic control towards objective treatment goals such as mucosal healing. There are seven monoclonal antibody therapies excluding their biosimilars approved by the US Food and Drug Administration for induction and maintenance of clinical remission in IBD. Adverse side effects associated with almost all currently available drugs, especially biologics, is the main challenge in IBD management. Natural products have significant potential as therapeutic agents with an increasing role in health care. Given that natural products display great structural diversity and are relatively easy to modify chemically, they represent ideal scaffolds upon which to generate novel therapeutics. This review focuses on the pathology, currently available treatment options for IBD and associated challenges, and the roles played by natural products in health care. It discusses these natural products within the current biodiscovery research agenda, including the applications of drug discovery techniques and the search for next-generation drugs to treat a plethora of inflammatory diseases, with a major focus on IBD.

## 1. Introduction

Inflammatory bowel disease (IBD) includes ulcerative colitis (UC) and Crohn’s disease (CD). UC was first described in 1859 [1], and CD in 1932 [2]. Both UC and CD are chronic and debilitating diseases without a real cure. As of 2017, 6.8 million IBD cases were reported globally, with an increase in age-standardized prevalence rates from 79.5 per 100,000 population in 1990 to 84.3 per 100,000 population in 2017 [3]. More than 1.6 million people in the United States [4], 250,000 in the United Kingdom [5], 260,000 in China [6], and 85,000 in Australia are affected by IBD [7]. Over the past few decades, IBD was most dominant in the western world as both the rate of incidence and prevalence were higher compared to developing countries. Recently, however, IBD incidence has increased rapidly in many Asian countries [5] with consistently rising trends, particularly in Japan, Korea, Hong Kong, and mainland China [8]. In Asia, males between the ages of 20 to 39 years are affected more by CD, and there is also a higher prevalence of ileocolonic CD, which is not the case in western countries [9,10,11]. There is a lack of national registries in many developing Asian, African, and Latin American countries and, therefore, much less is known about the incidence and prevalence of IBD.

IBD causes substantial morbidity [12] and heavy productivity losses [13,14]. The increasing trends in the rate of incidence and prevalence of IBD and lack of a cure or effective long-term treatment options have resulted in a substantial financial burden to the healthcare system worldwide [3,15,16]. When Park et al. [17] analyzed healthcare costs for 52,782 IBD patients in the United States on a per-year basis, IBD patients incurred a greater than 3-fold higher direct cost of care compared to non-IBD patients (US $22,987 vs. US $6956 per-member per-year paid claims) and more than twice the out-of-pocket costs (US $2213 vs. US $979 per-year reported costs), with all-cause IBD costs rising since 2013. Moreover, IBD patients have to bear significantly higher cost associated with the time they spend on their healthcare, unlike non-IBD patients. Similarly, Australia spends approximately AU $100 million per annum for IBD related hospitalization costs, more than AU $380 million related to productivity loss, and an additional $2.7 billion for other financial and economic costs [18]. The financial burden for IBD is highest during the first year of diagnosis, followed by a more stabilized cost pattern by seven to eight years after diagnosis. However, treatment costs tend to rise again after this period [17]. The fluctuation in the financial burden can be attributed to either age-related or healthcare-related factors, including inconsistent access to health care, support and education, and insecure funding for IBD-treating hospitals. Treatments (biologics, opioids, or steroids), emergency department use, and health care services associated with relapsing disease, anemia, or mental health comorbidity are some of the critical factors that may cause financial burden to all countries until a cure for IBD is established [17].

Despite the huge financial cost to the health care system and the morbidity burden faced by many countries, effective treatments for IBD remain elusive for many reasons. Recent reviews by Jeong et al. [19], Neurath [20], Kaplan [15], Ananthakrishnan [21], and Ruel et al. [22] highlight the epidemiology and risk factors for IBD, associated global health burden, and current therapeutic targets. There is, however, limited review coverage of the role of natural products in treating IBD. This current scoping review explores the causative factors, challenges in current treatment options, and the status of drug discovery from natural products for combating IBD.

## 2. Diagnosis and Pathophysiology of Inflammatory Bowel Disease

**Figure 1:**
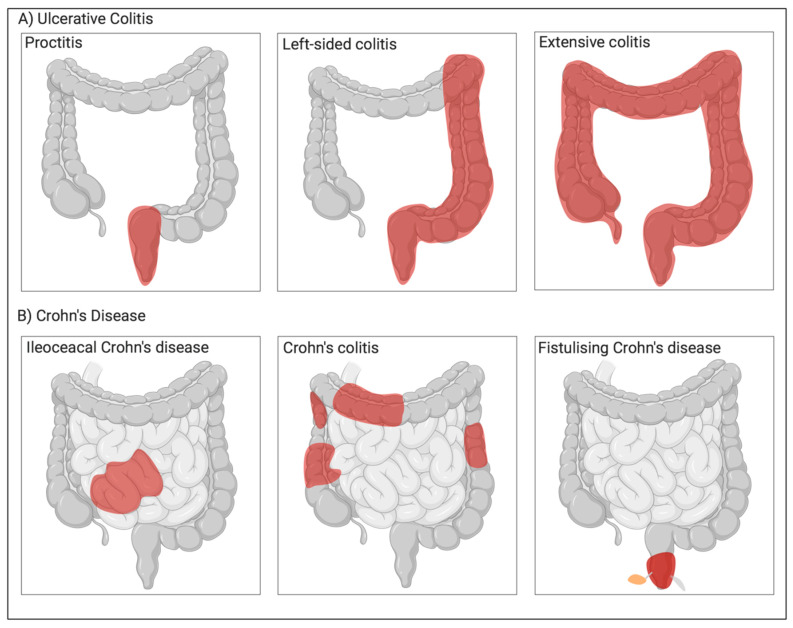
Different types of Inflammatory Bowel diseases. (**A**) Ulcerative colitis: proctitis, left-sided colitis, and extended colitis; (**B**) Crohn’s disease: Ileocecal Crohn’s disease, Crohn’s colitis, and Fistulising Crohn’s disease. Red indicates area of inflammation.

Both CD and UC show heterogeneity in many clinical and pathological features. They are distinguishable by their location and nature of inflammation (Figure 1). Unlike UC, which attacks colonic mucosa, CD can affect any part of the gastrointestinal (GI) tract [23]. Both conditions share clinical features like extra-intestinal manifestation, but hematochezia and passage of mucus or pus are common only in UC. Fistulas, perianal disease, colonic and small bowel obstruction is common in CD. Cryptitis and crypt abscesses are observed in both UC and CD, while crypt architecture is more distorted in the case of UC [24]. Both UC and CD show relapsing intestinal inflammation. Intermediate colitis (IC) sometimes does not present distinct clinical features of either UC or CD, particularly in colectomy specimens, rendering it hard to distinguish UC from CD. Although IC is not a unique disease or distinct clinical entity, it accounts for around 10% of the total IBD cases involving the colon [25], and this figure has not changed over the last 30 years [26]. Currently, IC is usually diagnosed when a distinction between UC and CD becomes difficult. A standard positive diagnostic test for IC is not yet available.

### 2.1. Ulcerative Colitis

**Figure 2:**
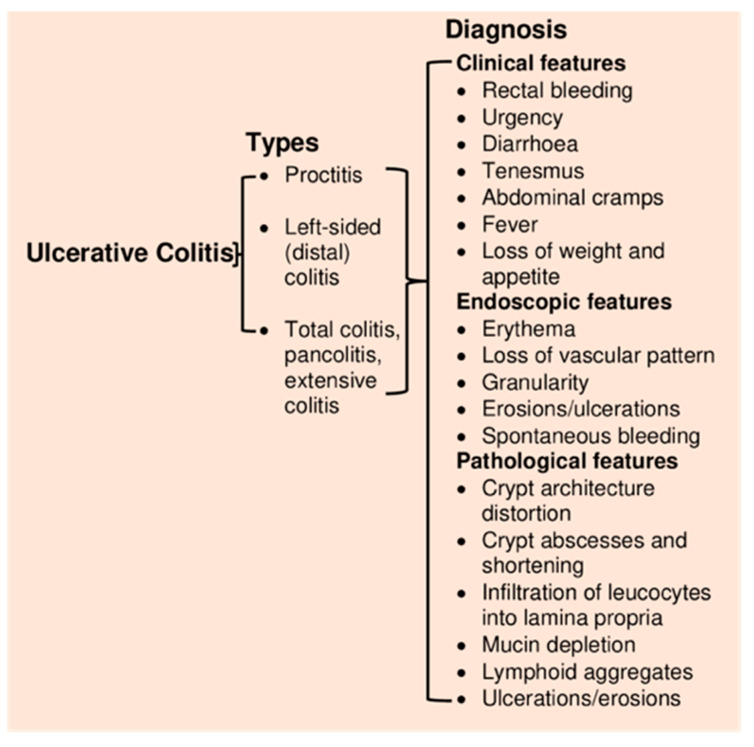
Types of ulcerative colitis and diagnosis.

UC causes inflammation and ulceration of the inner lining of the colon and rectum (the large bowel). There is no gender predominance in UC, and the peak age of disease onset is between ages 30–40 years [27]. Symptoms usually include diarrhea, abdominal pain, fatigue, loss of appetite and weight, and anemia. The severity of symptoms depends upon how much of the colon is affected or inflamed. Inflammation in UC is non-specific and may occur in the rectum (proctitis), left semicolon (left-sided colitis) or entire colon (extensive colitis or pancolitis) (Figure 2) [28].

Currently, there is no ‘gold standard’ test to diagnose UC [29]. Diagnosis of UC relies on test results obtained by colonoscopy, histopathology, blood testing, fecal examination, and radiological studies [30]. Patients diagnosed with UC at an older age are at lower risks of colectomy than those diagnosed at a younger age (below 16 years) [30]. According to a meta-analysis of population-based cohort studies, the risk of developing colorectal cancer is 2.4-fold higher in patients with UC, and the risk is higher in males than females and for those diagnosed with UC and extensive colitis at a young age [31].

**Figure 3:**
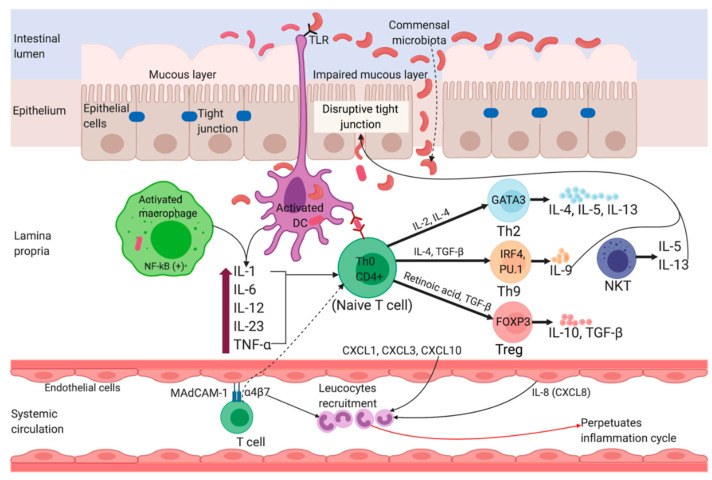
Pathophysiology of Ulcerative Colitis. Impairment of tight junctions and the mucous layer leads to increased permeability of the intestinal epithelium, resulting in more uptake of luminal antigens. Antigen presenting cells (APC) become activated upon recognizing non-pathogenic bacteria (commensal microbiota) through Toll-like receptors (TLRs). Activated APC initiate differentiation of naïve CD4^+^ T-cells into Th-2 effector cells (which produce pro-inflammatory cytokines such as TNF-α, IL-5, IL-6, and IL-13). TNF-α and IL-1 activate nuclear factor κB (NF-κB) pathway, which facilitate expression of pro-inflammatory and cell survival genes. Binding of integrin-α_4_β_7_ bearing T cells to the mucosal adhesion molecule MAdCAM-1 facilitate entry of more T cells into the lamina propria. Recruitment of circulating leucocytes due to the upregulation of inflammatory chemokines (chemokine ligands: CXCL1, CXCL3, CXCL8 and CXCL10) perpetuates the inflammatory cycle. MAdCAM-1, mucosal addressin cell adhesion molecule-1; IL, interleukin; TNF-α, tumor necrosis factor-alpha; TGF-β, transforming growth factor-beta; NKT, natural killer T; DC, dendritic cell; Th, T helper; GATA3, GATA binding protein 3; IRF4, interferon regulatory factor 4; PU.1, purine-rich PU-box binding protein; FOXP3, Forkhead box protein 3.

The pathogenesis of UC and CD is considerably distinct from each other. The change in luminal microbial diversity (dysbiosis), impairment of epithelial, and mucus layer barrier via disruption of tight junctions are strongly implicated in the pathogenesis of UC. Figure 3 shows an overview of the pathophysiology of UC. Although UC patients exhibit lower diversity of Firmicutes and a higher proportion of Gamma-proteobacteria and Enterobacteriaceae [32], and sulfite reducing deltaproteobacteria [33], whether such changes are intestinal inflammation-driven or vice versa remains controversial. Interleukin (IL)-13 produced by T helper type 2 (Th2) cells and non-classical natural killer T cells (NKT cells) also mediates UC [34,35,36] as it synergizes with tumor necrosis factor alpha (TNF-α) to regulate the expression of genes responsible for the formation of tight junction entero-epithelial cells [37]. IL-13 also disturbs the membrane integrity by increasing the rate of cell apoptosis (which intensifies upon exposure to TNF-α), and by changing the protein composition of the tight junctions [38]. An impairment of tight junctions increases gut permeability, leading to an enhanced influx of luminal antigens. Antigen-presenting cells (APC) such as macrophages and dendritic cells become activated upon recognizing non-pathogenic bacteria (commensal microbiota) through Toll-like receptors (e.g., Toll-like receptor 2 (TLR2) and TLR4) [39]. Activated APCs in turn initiate differentiation of naïve CD4^+^ (cluster of differentiation 4) T-cells into different subsets of effector T helper cells such as Th2, Th9, and regulatory T cells (Treg) in UC. In the inflamed lamina propria of UC patients, the expression of IL-4, which is a signature cytokine of Th2 cells, is dominated by high-level expression of other Th2-associated cytokines such as IL-5 and IL-13, and the Th2 master transcription factor GATA binding protein 3 (GATA3) [40]. Thus, UC is predominantly a Th2-mediated immune disorder, but considering the low-level expression of IL-4, the role of Th2 cells as a whole in UC remains inconclusive. IL-9-producing Th9 cells are also associated with UC as they prevent mucosal wound healing and disrupt protective functions of the mucus layer [41].

High levels of mucosal addressin cell adhesion molecule-1 (MAdCAM-1) causes increased recruitment of gut-associated lymphocytes to healthy gastrointestinal tract and sites of inflammation, implicating its role in the pathogenesis of IBD. Increased expression of MAdCAM-1 in the endothelium of the inflamed colon of rats induced by peptidoglycan-polysaccharide, and subsequent attenuation of colitis by anti-MAdCAM-1 antibody, imply a vital role of this molecule in the development of colitis [42]. MAdCAM-1 is equally abundant in the inflamed mucosa of both UC and CD, but MAdCAM-1+ venules are more abundant in the deeper layers of intestinal tissue from CD patients compared to UC (*p* < 0.001), and might account for the unique transmural inflammation in CD [43].

The mortality among the UC patients results mainly from comorbid conditions such as infectious and cardiovascular disease, and colon and biliary tract cancers [44]. Postoperative complications and comorbidity are additional factors for increased mortality within the first 2 years after diagnosis among patients >50 years of age and those diagnosed with extensive colitis [45].

### 2.2. Crohn’s Disease

**Figure 4:**
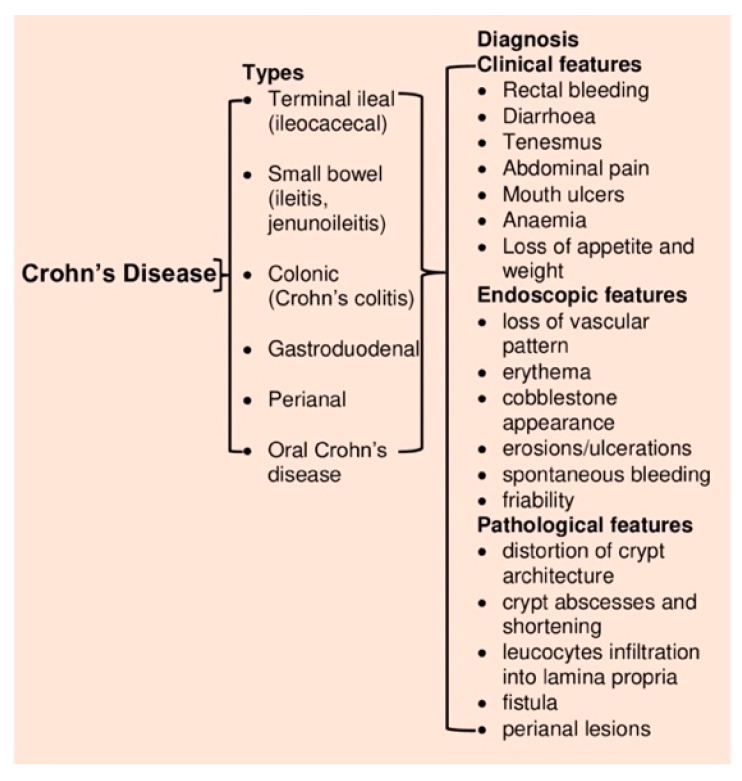
Types of Crohn’s disease and diagnosis.

CD is a chronic inflammatory disorder of the gastrointestinal tract affecting most commonly the terminal ileum, caecum, perianal area, and colon. Symptoms of CD are often insidious, making diagnosis difficult. However, symptoms such as chronic or nocturnal diarrhea, abdominal pain, bowel obstruction, weight loss, fever, or night sweats are critical parameters for initial diagnosis [46] besides other endoscopic or pathological features (Figure 4).

Delayed diagnosis of CD causes increasing bowel damage, fibrosis, and disability. Besides clinical assessment, blood- and fecal-based biomarkers are also used as an additional tool by clinicians to distinguish IBD from non-inflammatory diarrhea and for their management. For example, biomarkers such as fecal calprotectin (FC) is useful for screening IBD patients for endoscopic evaluation, and fecal lactoferrin in assessing the course of disease activity and healing [47,48,49]. And FC measurement is considered a promising non-invasive tool for clinical management of IBD. Although the choice of the optimal cut-off value for FC measurement remains a concern, higher FC cut-off value is known to maximize the accuracy of the diagnosis of IBD, particularly in UC compared to CD [50]. We did not elaborate on the role of FC in the clinical management of IBD, as it is reviewed in-depth by Mumolo et al. [51].

**Figure 5:**
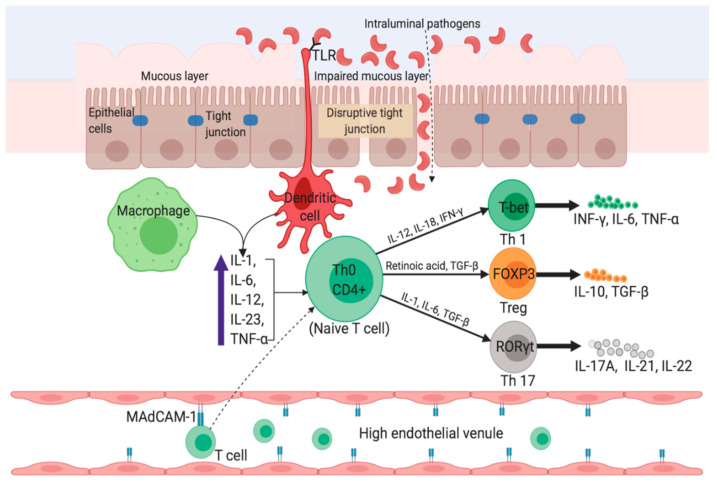
Pathophysiology in Crohn’s disease. The uptake of luminal microflora stimulates APCs (e.g., dendritic cells and macrophages) which in turn produce proinflammatory cytokines such as TNF-α, IL-6, and IL-23. Activated APCs facilitate subsequent differentiation of naïve CD4^+^ Th cells into Th1 and Th17 via expression of master transcription factors. Inside the high endothelial venule, binding of α_4_β_7_-bearing lymphocytes to MAdCAM-1 causes entry of more T cells into the lamina propria. IFN-γ, interferon-gamma; FOXP3, Forkhead box protein 3; RORγt, retinoic acid receptor-related orphan nuclear receptor gamma.

CD is a Th1 cell-mediated disorder. Small bowel inflammation in CD exhibits an increased level of proinflammatory cytokines such as interferon-gamma (IFN-γ) and IL-17A (produced by Th1 and Th17 cells, respectively) [35]. Moreover, the Th17 pathway (mediated by Th17 cell-derived IL-17) in turn influences the Th-1 response [52]. IL-6, IL-23, and transforming growth factor-beta (TGF-β), which are produced by innate immune cells and APCs, influences the IL-17 pathway (Figure 5). The high-level expression of transcription factors (e.g., STAT4 and T-bet) and cytokine receptors (e.g., IL-12Rβ2) promotes Th1 cell differentiation, which is characteristic of inflamed lamina propria of CD patients [53]. IL-12 derived activated APCs stimulates the Th1 master transcription factor T-bet [54]. The expression of IL-23 by ileal dendritic cells stimulates IL-17 production, and as a result, both IL-17 and IL-23 are present in elevated levels in CD patients [54]. Thus, the development of CD is associated with both Th-1 and Th17 pathways. The mortality rate in CD is mainly due to pulmonary disease and cancers of the biliary tract, lymphoid and hematopoietic tissues [44].

## 3. Causes and Risk Factors of Inflammatory Bowel Disease

**Figure 6:**
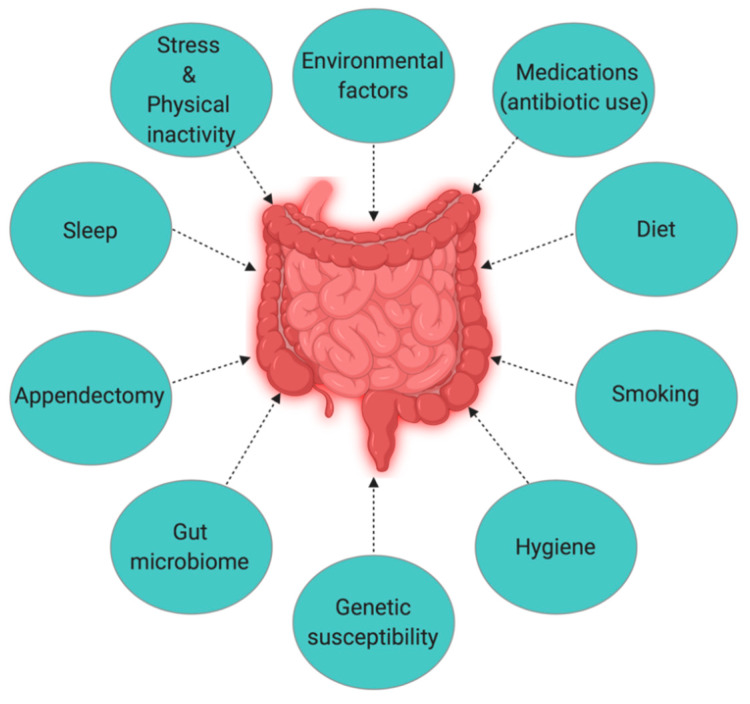
The interplay of factors causing inflammatory bowel disease (IBD).

The Hygiene Hypothesis is a central theme to the growing incidence of IBD, but it is still difficult to pinpoint which particular factors are responsible for causing IBD. Strachan first proposed the Hygiene Hypothesis in 1989 to explain the increasing incidence of atopy (allergic disorders) [55]. Later, many authors claimed through epidemiological studies and various experimental models that autoimmune disorders could be a result of broad environmental, infectious burden rather than individual behavior/hygiene [56,57,58,59]. According to the Rook’s reinterpretation of hygiene hypothesis (Old Friends Hypothesis as proposed in 2003), immunoregulatory disorders would occur first in those individuals with reduced/minimal contact with pathogens including commensal microbes and helminths (old friends) that are known to prime immunoregulation (Treg activity) in the human gut [60]. The rapid rise in the incidence of IBD over the last century in both developing and developed countries [3,15] could be related to improved hygiene practices such as access to clean water, non-contaminated food, and reduced family size [61]. While the definitive cause of IBD remains elusive and unknown, many studies point to 10 different causative factors (Figure 6) [23], with three major factors being genetic, environmental, and diet (which influences the host’s gut microbiota).

### 3.1. Genetics

Genome-wide association studies identified 163 susceptible gene loci in IBD (with 110 common, 30 CD specific, and 23 UC specific) [62], highlighting the role of genetics in IBD pathogenesis. However, more than 50% of IBD susceptible gene loci are also associated with other inflammatory and autoimmune diseases [63]. MDR1 (multidrug resistance 1) gene in human chromosome 7 is one such gene that is associated with the pathogenesis of UC [54]. A comprehensive understanding of the mechanisms of IBD pathogenesis induced by genetic factors requires studying the role of an individual gene, which can be a challenging task. However, less than 50% concordance for IBD in twins represents the significant role that environmental factors might have in the development of IBD [64]. Another study conducted by Thompson et al. in British twins also reported similar (only 17% concordance for IBD) results among identical twins [65]. Moreover, the concordance between two disease conditions (CD and UC) among twins is not the same. Analysis of a Swedish twin cohort showed higher concordance (50%) for CD than for UC (18.8%), suggesting that genetic influence or heritability is higher in CD than in UC [64,66,67].

### 3.2. Environmental Factors

After extensive studies trying to determine the role of genetics in the pathogenesis of IBD have not yielded adequate evidence, many assume environmental factors to have a more significant role than genetics. Numerous studies have analyzed the linkage between various environmental factors and the development of IBD. When Khalili et al. studied the association of geographical variation with the incidence of IBD (both CD and UC) in two large prospective cohorts of US women (175,912), they found a higher incidence of both CD and UC among women residing in the northern latitudes, which could be attributable to less exposure to sunlight or ultraviolet B (UV-B) radiation [68]. UV-B radiation induces UV-Treg cells that have the potential to suppress inflammatory responses [69]. People living in higher altitudes are also more likely to have vitamin D deficiency due to insufficient exposure to sunlight [70]. Lack of vitamin D is also considered a possible cause of IBD since vitamin D receptor knock-out mice develop severe inflammation [71].

Numerous studies are suggesting a role for the environment in the pathogenesis of IBD, notably from a migration perspective [72,73,74]. For instance, children of immigrants who arrived in Canada at a younger age have an increased risk of IBD [75]. While Zoetendal et al. compared the risk of developing IBD among African-Americans and Africans inhabiting semi-urban (westernized) and rural environments respectively, the risk was significantly lower among those who were born and raised (first five years) in unhygienic environments like livestock farms compared to those living in the city [76]. Selective exposure to various environmental factors or conditions is also responsible for determining the microbiome composition. A study carried out in South Africa observed distinct gut microbiome composition in genetically similar populations in rural areas versus urban areas [77], where rural subjects contained significantly lesser Bacteriodetes populations than semi-urban and urban subjects. While a similar comparative study from India showed a dominance of phyla Bacteriodetes and Proteobacteria in the gut microbiome of rural subjects, phyla Firmicutes, and Lactobacillus in urban subject [78]. These findings indicate that exposure to different environmental conditions/lifestyles can influence the microbiome composition among a similar population.

### 3.3. Microbiota

The commensal human gut microbiota is essential for maintaining intestinal epithelial homeostasis and protection from mucosal injury [79]. The human lower GI tract has 10^14^ microbial cells [80], and Bacteriodetes and Firmicutes are the two most dominant phyla in the gut [81]. The gut microbiome determines the normal functioning of human health. For example, fibrinolytic bacteria degrade polysaccharides in the gut into smaller carbohydrates and short-chain fatty acids [82], and microbiota of the lower GI tract use dietary fiber as a source of energy [83]. Any changes in the composition of the healthy microbiota (dysbiosis) in the gut can trigger abnormal inflammatory responses. Firmicutes species, such as *Faecalibacterium prausnitzii*, has been reported to be poorly represented in patients with active IBD compared to healthy subjects [84]. The study conducted by Martin et al. further supports the protective role of this species, where intragastric administration of *F. prausnitzii* significantly decreased the severity of colitis in the trinitrobenzene sulfonic acid (TNBS)-induced mouse model of colitis [85]. The host must maintain a balance between recognizing pathogenic from commensal microbial species, as any disturbance in the composition of commensal species could trigger abnormal inflammatory responses such as IBD.

### 3.4. Diet and Smoking

Diet influences the composition of the microbiota and their metabolic activity in the human gut [86]. There is a growing concern that the western diet, rich in fats and sugars, is responsible for the change in the diversity and metabolic activity of human gut microbiota, thereby contributing to the increasing incidence of IBD [87,88]. The increase in the abundance of *Bilophila wadsworthia* due to an animal-based diet can facilitate the growth of microorganisms that can trigger IBD [87,88]. Moreover, *B. wadsworthia* also produces hydrogen sulfide that can cause damage to intestinal tissues [86]. Long-term dietary pattern influences the development of IBD [89]. For instance, the intake of fruits decreases the risks of developing CD [90], although the underlying mechanism is yet to be understood. Smoking is one of the contradictory factors linked to IBD. While smoking is harmful to CD patients, reports show beneficial in UC [63]. The positive effect of smoking in UC is evident from the “Boston Drugs Surveillance Program” [91], ”UC patients in Birmingham, England” [92], and ”Oxford Family Planning Association Contraceptive Study” [93]. Additionally, the transdermal treatment of active UC patients with nicotine patches also showed better remission compared to the placebo group [94]. However, it is still controversial, and more research is required to determine if nicotine is one of the active components of cigarette smoking that is responsible for the beneficial effects on the UC disease course.

### 3.5. Sleep Deprivation, Stress, and Physical Inactivity

Inadequacy of sleep and psychological distress are additional intrinsic factors known to associated with inflammation and the inflammation system. Sleep disturbances are said to be common in IBD patients [95,96]. Some studies [97,98] have reported that symptoms of depression and anxiety cause clinical recurrence in IBD patients. However, stressful life events are not associated with the onset of inflammatory disease [99]. Alteration of sleep pattern or circadian rhythms [100] and insufficient sleep (<6 h/day) [96] has a direct impact on disease course and severity. A study involving 136 Japanese IBD patients found sleep disturbances as a potential risk factor of disease flare-up for both UC and CD within one year [95], but a similar kind of study (3173 IBD patients with sleep disturbances) conducted by Ananthakrishnan et al. [101] could observe an increased risk of disease flares only in CD within 6 months. A positive correlation between psychological distress and IBD flare-ups [102,103] indicates the need for timely psychological therapy in IBD patients.

### 3.6. Appendectomy

Appendectomy (i.e., surgical removal of the appendix) and its association with the development of UC and CD is a scarcely explored area of research [104]. Few studies involving both humans as well as animal models showed evidence for a role of the appendix in gastroenterology. T-cell receptor-α mutant mice (TCR-α^−/−^) appendectomized at a young age (3–5 weeks old) contained more mesenteric lymph node (MLN) cells compared to the placebo group (sham-operated TCR- α^−/−^ mice), indicating that the appendix could be an important site for priming MLN cells involved in causing IBD [105]. Similarly, Mombaerts et al. also found that an increase in the number of MLN cells in TCR-α^−/−^ mice is related to the development of IBD [106]. A few studies and case reports have also shown the positive effect of appendectomy on the clinical course of UC in human subjects. A study of IBD patients in Australia confirmed that appendectomy before diagnosis delays disease onset of both UC and CD and results in fewer flare-ups in the case of UC when compared with patients without prior appendectomy [107]. A case report from Korea also confirmed that a patient with UC experienced a more extended period of remission after appendectomy [108]. However, the therapeutic relationship between CD and appendectomy remains inconclusive. [30].

### 3.7. Antibiotic Use

A leading hypothesis in the etiology of IBD is the alteration in the human gut microbiota that triggers abnormal inflammatory responses, including IBD. Multiple factors are assumed to be responsible for inducing gut dysbiosis. Childhood exposure to antibiotics is one among them [109]. Children exposed to antibiotics at an early stage [109,110,111,112] and adults who had medication for acute gastroenteritis [113] possess higher risks for IBD. The frequency of use of antibiotics and the age at the time of use may have a varying effect as risks for IBD tend to decrease with increasing age at the time of exposure [114]. Regular intake of non-steroidal anti-inflammatory drugs like aspirin showed a strong positive correlation with only CD [115].

## 4. Current Treatment Options for IBD

### 4.1. Conventional Treatments

**Figure 7:**
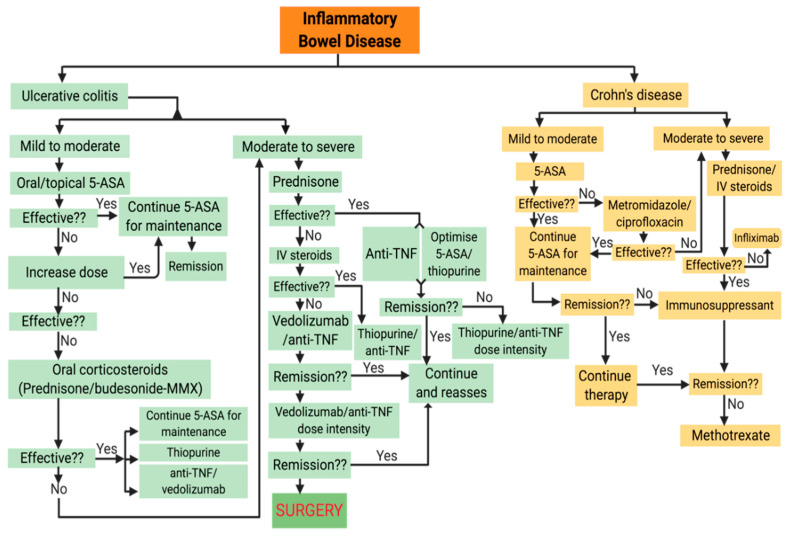
Current treatment options for Inflammatory Bowel Disease (both ulcerative colitis (UC) and Crohn’s disease (CD)).

Prompt diagnosis and identifying the specific treatment goals (e.g., mucosal healing) can help to create better long-term outcomes for patients. As there is no “gold standard” to define disease severity; thus, only working definitions of disease activity are available to help guide IBD therapy. Figure 7 below, adapted from Ordás et al. [116] and Knutson et al. [117], presents an overview of currently available treatment algorithms for UC and CD. The main conventional treatments for IBD are biologics, oral corticosteroids, and salicylates, discussed in the subsequent sections.

#### 4.1.1. Small Molecule Drugs

Small molecules represent a potential source of selective drugs in IBD. Many small molecule drugs (SMDs) such as corticosteroids (e.g., budesonide, prednisone), immunomodulators (e.g., azathioprine, 6-mercaptopurine, and methotrexate), and aminosalicylates (e.g., sulfasalazine and mesalazine) came into clinical practice as early as 1955 for IBD [118,119]. SMDs (mol. Wt. <1000 Da) have stable structures, and their cost of production is also comparatively cheaper than biologics. The shorter half-life of SMDs is another advantage, particularly during situations like infection, surgery, and pregnancy when rapid elimination of the drug is required [120]. Moreover, unlike biologics requiring the parenteral route for administration, oral SMDs can boost a patient’s satisfaction and enhance treatment adherence and efficacy [121].

The SMD tofacitinib [122] has recently proven to be safe and effective in the induction and maintenance of clinical remission in UC. During phase 3, randomized, double-blind, placebo-controlled trials of tofacitinib therapy in 905 adults with moderate to severe UC, approximately 18% achieved clinical remission in 8 weeks as compared to 6% in the placebo group patients [123]. Tofacitinib under two different trade names (XEL JANZ^®^ (Pfizer Inc., Groton, NY, USA) and XEL JANZ XR^®^ (Pfizer Inc., Groton, NY, USA) appeared in the market since 2018 as the first drug of the Janus-kinase (JAK) inhibitor class, and it has displayed efficacy against moderate to severe UC with an excellent safety profile [124]. Aminosalicylate (5-ASA) and its derivatives, such as mesalamine [122] is another excellent example of SMD in the therapeutic management of IBD. However, some SMDs, including corticosteroids, are associated with adverse side effects. Sulfasalazine is associated with allergic and dose-dependent intolerant side effects [125]. Besides the SMDs mentioned above, there are 22 additional SMDs under clinical development for the treatment of IBD, including JAK-inhibitors, sphingosine-1-phosphate receptor modulators [121].

#### 4.1.2. Biologics

Biologics, or biological agents or biological response modifiers, are a group of molecules, including recombinant cytokines, monoclonal antibodies, and specific antagonists of cytokines and soluble receptors that are involved in modulating inflammation during immune-mediated processes. Biologics are more immunogenic than SMDs [126]. They are optional treatments for patients with poor or no response to other drugs such as immunosuppressants (azathioprine, mercaptopurine, and methotrexate) or steroids, or patients suffering from strong side effects of other IBD drugs. The US Food and Drug Administration (FDA) has so far approved seven protein-based therapies (monoclonal antibody therapy) for IBD treatment, namely vedolizumab, natalizumab, adalimumab, infliximab, golimumab, certolizumab pegol [127] and ustekinumab (Table 1). Vedolizumab, the first anti-integrin approved for use in UC, is known to be the safest biologic available with minimal side effects [128]. It binds to α4β7 integrin (present on the surface of intestinal endothelial cells) and prevents the migration of leukocytes into the gut. Leukocyte infiltration into the gut mucosa via interaction between integrins on lymphocytes and their ligands on endothelial cells of intestinal lymphoid tissue (MAdCAM-1) is another major event in the pathogenesis of IBD [129,130]. Inhibition of leukocyte migration is possible by blocking the integrin-MAdCAM-1 interaction. Anti-adhesion agents such as natalizumab, which blocks the α4 subunits of integrins on lymphocytes, can be an option for IBD maintenance but is likely to be associated with increased risk for specific side effects [131]. From a comparative meta-analysis on the efficacy of approved biologic agents, adalimumab is better than certolizumab pegol, vedolizumab, and infliximab in both induction and maintenance of CD, while in UC, infliximab is better than adalimumab and golimumab [132].

Cytokine-targeted therapy is another treatment option that has transformed the treatment of IBD [124,133]. Identifying specific cytokines involved in developing particular features and phases of IBD has enabled the design of better treatment options. Cytokines play a vital role in controlling intestinal inflammation and the associated clinical symptoms of IBD. But some of the cytokines possess dual functions (both pro-and anti-inflammatory), which presents difficulties in designing specific cytokine-based therapeutic drugs. For instance, IL-6 pathway inhibition is helpful in the successful treatment of rheumatoid arthritis, while IL-6 produced by intraepithelial lymphocytes following the onset of inflammatory injury promotes epithelial proliferation and wound repair in murine models of bowel injury [134]. IL-1 family cytokines such as IL-1α produced by epithelial cells are inflammatory, while IL-1β produced by myeloid cells promotes healing and repair [135]. Thus, the development of specific cytokine-based therapies in the future is crucial but also challenging. Even though this therapy can control symptoms and prolong the relapse-free period for some patients, many patients fail to respond [136]. For example, 30% of the patient shows primary non-response and 13-25% secondary non-response with anti-TNF-α therapy [137,138].

Although the use of biologics in developed countries is common, most of these drugs are yet to reach in developing countries. Higher manufacturing and quality control costs make biologics very expensive, and thus affordability of such drugs by many developing countries is questionable [139]. So, introducing biologic therapies for IBD is going to impose a burden on the healthcare system of developing countries [140]. Development of biosimilars that can substitute original biologics with minimal treatment cost is urgent; otherwise, IBD medications may continue to remain far from the hands of economically disadvantaged patients. Biosimilars are drugs developed with maximum similarity to their original products of biologics in terms of purity, safety, and efficacy. Six biosimilars including three infliximab biosimilars-Inflectra^™^ (infliximab-dyyb, CELLTRION, Inc. Yeonsu-gu, Incheon, Korea), Renflexis^™^ (infliximab-abda, Samsung Bioepis Co., Ltd, Incheon, Korea), and Ixifi^™^ (infliximab-qbtx, Pfizer Ireland Pharmaceuticals Ringaskiddy, Co. Cork, Ireland), three adalimumab biosimilars–Amjevita^™^ (adalimumab-atto, Amgen Inc., Thousand Oaks, CA, USA), Cyltezo^™^ (adalimumab-adbm, Boehringer Ingelheim Pharmaceuticals, Inc., Ridgefield, CT, USA), and Hyrimoz^™^ (adalimumab-adaz, Sandoz Inc., Princeton, NJ, USA) are already approved by the US FDA for use in IBD as of 2018 [126].

Several new therapeutic strategies that are currently in clinical trials include recombinant anti-inflammatory cytokines (IFN-γ, IL-10, IL-11) and inhibitors of cell adhesion molecules (natalizumab and etrolizumab), anti-interleukin-12/23 (ustekinumab), pro-inflammatory cytokine (IL-12), and their receptor (IL-6R) [165,166,167,168]. Besides recombinant anti-inflammatory cytokines and inhibitors, there are few existing drugs repurposed for treating IBD. For instance, an anti-mycobacterium drug RHB-104 (product of RedHill Biopharma Ltd based in Tel-Aviv, Israel) was repurposed for CD, and it is in phase III trial [169]. Adacolumn is another example of an emerging treatment for IBD, which is a safe, non-drug intervention. Adacolumn absorbs excess neutrophils and CD14^+^ CD16^+^ monocytes (which produces TNF), and these absorbed granulocytes/monocytes, in turn, releases interleukin receptors such as IL-1 receptor antagonist, hepatocyte growth factor and soluble TNF receptors possessing anti-inflammatory properties [170]. This treatment showed highly positive results against UC in a few countries, including Japan and the United Kingdom [171,172,173].

#### 4.1.3. Surgical Treatment

Surgery would cause extensive loss of small bowel and disability, including permanent ostomies [174], and it is usually performed as a last choice of treatment options, especially in a complicated situation when prolonged medication would cause more significant disability than surgical alternatives. Surgery in UC, unlike CD, is a curative therapy since the disease is limited to colon and rectum, while in CD, it can only control the complication of the disease process [175]. Despite the introduction of anti-TNF drugs, about 10–30% of UC patients ultimately require a colectomy for the management of dysplasia or cancer [176]. While there are reports on the decreased rate of early surgery in CD patients after treatment with anti-TNF drugs [177], as only one-third of CD patients require surgical therapy within the first five years of their diagnosis [178].

The choice of surgery depends upon advantages as well as disadvantages associated with each surgical option. For instance, for UC patients, restorative proctocolectomy with ileal pouch-anal anastomosis (IPAA) is considered as standard surgical treatment, as it can maintain a usual route of defecation by linking ileal pouch to the anal canal, and avoids ostomy permanently [179]. But chronic pouchitis is still the main factor that limits the success of the surgical cure of UC [180]. In CD, since the almost entire colon is involved in the inflammation, surgical decision-making is more challenging compared to UC. It depends upon the extent and location of the inflammation in the entire colon. If the right side of the colon is affected, right colectomy or extended right colectomy with ileocolic anastomosis is an option. When the left colon or entire colon is affected, particularly in Crohn colitis, total abdominal colectomy with ileorectal anastomosis is performed [181]. Surgery is not a cure for CD since almost 28% of patients experience small bowel clinical recurrence after total colectomy with permanent ileostomy [182]. Besides clinical recurrence, post-operative complications is another challenge in the surgical treatment of IBD. However, due to the current laparoscopy-assisted surgery approach, IBD patients experience improved cosmesis, reduced intraoperative blood loss, and early recovery [183], which is lacking in open surgery. Ahmed et al. [184] and Beyer-Berjot et al. [185] also reported that laparoscopic IPAA does not affect fecundity in females. Surgical treatment procedures and options for both CD and UC are covered elaboratively in the guidelines developed by the European Crohn’s and Colitis Organisation and European Society of Colo-Proctology [183,186].

While the attitude of many IBD patients on surgery as the last option remains unchanged, few chronic patients believe that it is better to get it done ‘once and for all’ through surgical procedures because of immense and ineffective drug therapies. It is, therefore, treated as an ‘alternative treatment strategy’ instead and ‘last choice of treatment options.’ IBD is elusive, and it is often confused for irritable bowel syndrome (IBS), which can complicate surgical treatments if based on the wrong diagnosis. It is challenging to low-resource developing countries where there is a lack of expertise and surgical technologies. This factor often influences the decision of health workers and IBD patients. There is a need to provide training for health workers in developing countries and step-up the testing to obtain accurate clinical numbers. The top-down approach, where government taking initiatives and providing advocacy to educate the public would be an excellent strategy to capture the actual IBD incidence worldwide, especially in developing countries.

### 4.2. Alternative Treatment Options for IBD

It is reported that about 21–60% of IBD patients have used complementary and alternative medicine, which includes herbal therapy, mind-body intervention, and acupuncture treatment [187,188,189]. Herbal therapy and acupuncture are the two most popular treatments used by both UC and CD patients [189]. A lack of well-designed studies and randomized, double-blind clinical trials have resulted in the safety and efficacy of complementary and alternative medicine continues to be questioned [189].

#### 4.2.1. Botanicals Used for Treating IBD

Botanicals are popular among IBD patients owing to their perceived safety (i.e., lack of side effects) and efficacy [190]. Amongst the herbal drugs or botanicals, *Plantago ovata, Curcuma longa, Andrographis paniculata, Aloe vera, Artemisia absinthium, Boswellia serrata, Cannabis sativa*, and *Tripterygium wilfordii* are the most popular herbal remedies used by IBD patients. A recent review by Triantafyllidi and colleagues (2015) on herbal therapies for the treatment of human IBD came across 27 clinical studies on 11 herbal therapies involving 1874 IBD patients. Of the 11 herbal therapies, seven of them had beneficial effects in UC, while four resulted in clinical remission in CD [190]. Nine out of 30 active UC patients showed clinical remission at 4 weeks after taking *A. vera* gel orally compared to one out of 14 patients in the placebo group [191]. *P. ovata* seeds, when compared with mesalamine, showed an equivalent effect on maintaining remission in UC after 12 months [192]. Curcumin isolated from *C. longa* is another botanical, which is used for UC. Both crude extract and a pure compound (andrographolide) isolated from *A. paniculata* showed efficacy against mild to moderate UC [193,194]. Similarly, *A. absinthium* and *T. wilfordii* seem to provide remission in CD [195]. In the USA, 22 states have legalized *C. sativus* for medicinal use, and approximately 15–20% of American IBD patients rely on cannabis extracts [196].

#### 4.2.2. Helminth Therapies

Helminths, most commonly referred to as parasitic worms, have successfully co-evolved with their human hosts through adopting a parasitic lifestyle. They can survive in the niche inside the human host by modulating the host’s regulatory immune network [197]. Helminth therapy using hookworms and whipworms has gained international attention. IBD patients have used these parasites for many years despite concerns around safety and regulatory issues. Preliminary studies on the beneficial effects of helminths in IBD were described by Elliott et al. when intra-rectal administration of TNBS failed to induce colitis in mice infected with the blood fluke *Schistosoma mansoni*. Oral administration of eggs of the whipworm *Trichuris muris* significantly reduced TNBS-induced colitis in IL-10−/− mice [198]. In terms of human trials, Summers and colleagues administered *Trichuris suis* ova to 29 patients with active CD at intervals of three weeks for 24 weeks, and 21 of the patients were in remission at week 2, 4 indicating a safe and effective alternative therapy for CD [199]. There have been more than 20 different clinical studies conducted in IBD using different helminths (*Trichuris suis* ova, *Necator americanus* infective third-stage larvae (L3)), and helminth-derived products (excretory/secretory proteins from *Schistosoma mansonii* and *Ancylostoma caninum*); this literature has been reviewed elsewhere [200,201]. However, *T. suis* ova therapy failed to show its clinical efficacy against active CD in stage 2 clinical trial [202].

### 4.3. Challenges in the Treatment Regimens and Management of IBD

Although the overall health of IBD patients and their quality of life has improved by the earlier diagnosis and new treatment therapies, there are still many challenges remaining unsolved. One of the main challenges within IBD treatment and management is almost all available IBD therapies are associated with many adverse side effects (Table 1). Optimization of diagnostic strategies and accessibility and affordability of new and emerging IBD therapeutic drugs are two of the many challenges discussed below.

#### 4.3.1. Diagnosis

Early and accurate diagnosis of IBD is crucial for better treatment outcomes. However, none of the serological and fecal diagnostic biomarkers offer a stand-alone tool for practical evaluation, both for suspected and established IBD. New approaches to discover biomarkers through gene expression studies including Affymetrix GeneChip technology in whole blood [203], in mucosal biopsies [204], mRNA expression levels in peripheral blood mononuclear cell RNA [205], and circulating miRNA [206] have shown exciting and promising results in preliminary studies. After diagnosis, most IBD drugs used for treatment do not produce clinical remission, or they are associated with numerous adverse effects, including diarrhea, renal impairment, and opportunistic infections including, tuberculosis [144,207,208,209,210]. Assessing inflammation using endoscopy is expensive as well as invasive. The initial approach of using combined information of phenotype and serology is not enough to distinguish CD from UC [211,212,213], hampering the accurate prediction of clinical outcome.

Metabolomics is emerging as a tool for comprehensive analysis of metabolites in any biological sample, and this technique could play a vital role in IBD diagnosis, drug target identification, and customized treatments (precision medicine) [214]. For example, the T1259 serum metabolite profiles can differentiate IBD from healthy subjects [215]. Similarly, serum and fecal metabolite analysis proved promising in differentiating pediatric IBD from healthy subjects via the level of FC and metabolites such as choline [216]. The fecal FC assessment is not only used in the pediatric setting; for instance, in UC patients, FC performs better than C-reactive protein in predicting clinical activity such as degree of inflammation and mucosal healing [51]. As stated earlier, the in-depth role of FC in IBD, including monitoring the effectiveness of therapy, predicting disease relapse, and postoperative recurrence, is available in the review by Mumolo et al. [51]. Metabolite profiling can also identify distinct biomarkers that can differentiate subtypes of IBD [217]. Analyzing metabolites using different statistical approaches can determine similarities as well as differences in metabolite profile that can differentiate disease phenotypes. For example, biopsies from patients with active UC contain elevated levels of glutamine and glutathione compared to control tissue or patients with inactive UC [218]. However, metabolomic techniques and spectroscopic tools are incredibly costly, and the affordability of such advanced facilities by developing countries remains questionable.

#### 4.3.2. Accessibility and Affordability of IBD Treatments

The availability and affordability of newly introduced drugs is another challenge in IBD management. Antibodies to human TNF-α inhibitors (anti-TNFs; infliximab, adalimumab, and certolizumab pegol) are costly drugs [219]. For example, 100 mg vial of infliximab would cost USD 549.70, whose initial prescription for CD patient is 5 mg/kg body weight, meaning a cost per infusion for a 73 kg patient comes to USD 2,198.18 and per annum USD 16,485.04/patient [220]. Similarly, for adalimumab, initial 80 mg and 40 mg after two weeks cost USD 1,405.63, and for one year, USD 12,176.45 if infused 40 mg weekly, but these costs may subject to change with currency fluctuations. The high cost of biologics has created disparities in usage among patients. Such disparities can be narrowed only by introducing affordable alternative therapeutics such as biosimilars, as stated in the earlier sections. Biosimilars are reference products easy to produce, unlike original biologics obtained from living organisms, and thus biosimilars are estimated to reduce the original spending on biologics by 3% over the same period [221].

Studies have shown that poor mental health is associated with disease flares and a generally poorer IBD prognosis [97,222]. But providing adequate mental healthcare access to IBD patients remains a hurdle even in many developed countries, including Australia. In one of the most extensive surveys focusing on mental health needs, attitudes, and access to mental health services in people living with IBD in Australia [98], a significant gap was identified in the mental healthcare services among IBD patients. Only 12% of the total of 731 participants had access to psychologists. Other studies [223,224,225] also reported similar findings where more than a quarter of hospitalized IBD patients are affected by mental distress. IBD patients suffering from depression are at higher risk of worsening disease due to non-compliance with treatment [226]. Thus, regular provision of mental health screening and reasonable access to psychologists or mental health professionals besides treatment could be another key strategy towards improving the quality of life of IBD patients, which can subsequently reduce the cost burden associated with disability.

## 5. Anti-Inflammatory Activities of Natural Products

**Figure 8:**
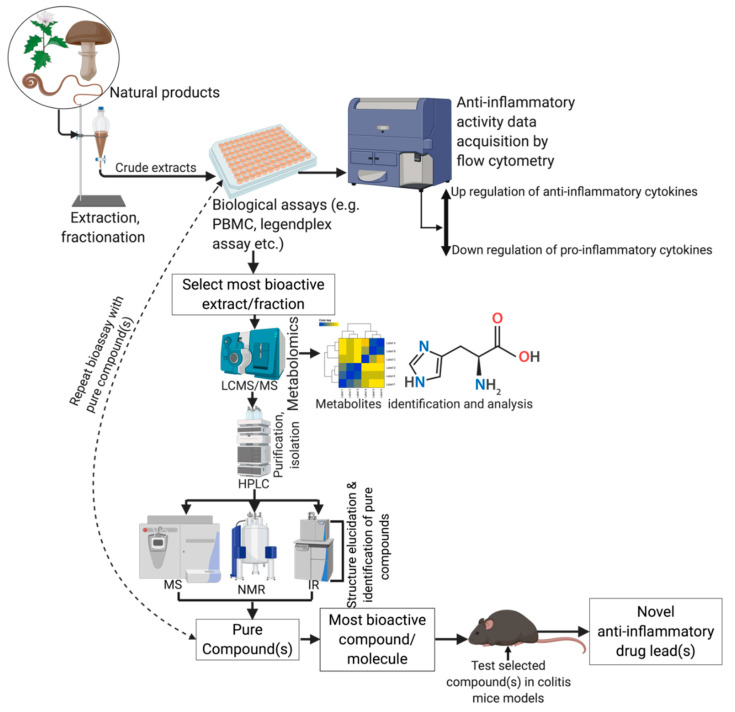
Schematic representation of the techniques used in anti-inflammatory drug-discovery from natural products. PBMC, peripheral blood mononuclear cell; LCMS, liquid chromatography mass spectrometry; MS; mass spectrometry; HPLC, high performance liquid chromatography; NMR, nuclear magnetic resonance; IR, infrared red.

Natural products, including plants, animals, fungi, microorganisms, and marine organisms produce secondary metabolites with rich structural diversity, which synthetic and combinatorial chemistry approaches are lacking, and for this reason alone, natural products are viable sources of drugs [227,228,229]. Four main bio-rational search strategies guide the identification of novel drug lead compounds from natural sources. They are: (i) ethnobotanically directed approach, (ii) ecologically directed approach, (iii) chemically directed approach, and (iv) random selection and screening (Figure 8) [230]. Very often, approaches (i), (ii) and (iv) are combined by researchers for efficiently discovering novel drug lead compounds.

Therapeutic agents derived from natural sources may be small molecules or biologics [231]. Small molecule natural products with anti-inflammatory properties hold great potential for clinical translation (Table 2). The Anti-inflammatory Compounds Database (freely accessible at http://956023.ichengyun.net/AICD/index.php.) has recorded over 79,781 small molecules of the therapeutic potential and a total of 232 inflammation-related targets [232]. Ten out of 2,892 natural products showed potent efficacy as anti-inflammatory drug lead candidates when assessed for their anti-inflammatory activity in the prediction model constructed using iterative stochastic elimination algorithm. However, of these 10 natural products, only three (moupinamide, hypaphorine, and capsaicin) have thus far been experimentally reported as an anti-inflammatory and require further study on the remaining seven [233].

### 5.1. Techniques and Biological Assays Used in the Discovery of Anti-Inflammatory Extracts and Drug Leads

‘Omics’ science such as genomics, proteomics, lipidomics, and metabolomics has enabled the comprehensive characterization of biomolecules of diverse fauna and flora, facilitating a better understanding of disease mechanisms. Of these ’omics’ approaches, metabolomics remains the least applied and under-utilized. A combined approach using metabolomic profiling and in vivo anti-inflammatory activity studies of identified metabolites in various animal models such as colitis models has given insights into disease mechanism and identifying potential therapeutic targets. For example, Gas Chromatography-Mass Spectrometry (GCMS)-based metabolomic profiling of dextran sulfate sodium (DSS)-induced colitis in mice had proven useful for monitoring disease progression and differentiating active from inactive colitis [234]. Furthermore, multivariate indexes developed based on Fischer linear classifiers from plasma amino acid profiles (aminogram) could be used as a discriminator between CD and UC patients as well as in monitoring disease activity during the progression of IBD [235]. Metabolomics techniques use Nuclear Magnetic Resonance (NMR), GCMS, Liquid Chromatography-Mass Spectrometry (LCMS), and hyphenated spectroscopies for bulk identification of compounds present in the crude and complex mixtures of natural products. These same spectroscopies, in combination with High-Performance Liquid Chromatography (HPLC), Infrared Red (IR), and X-Ray Crystallography, are used for compound purification and structure elucidation [230]. Pure compounds, once isolated, must be screened for their anti-inflammatory activities using different in vitro assays and in vivo disease models.

A comprehensive understanding of the complex disease mechanisms that underpin IBD is crucial for identifying targets for treatment, and subsequently to design novel drugs that hit these targets. The application of different murine models of IBD has profoundly improved the understanding of IBD pathogenesis and assisted in the development of novel therapeutic strategies. As of now, researchers use at least 66 different kinds of animal models to study IBD [236,237]. These disease models are chemically-induced, cell-transfer, congenic mutant, and genetically modified models. The use of genetically engineered mouse models that spontaneously develop colitis has enabled a deeper understanding of the complex immunopathogenesis mechanisms of IBD. The DSS–induced colitis model is one of the most widely used models since it is easy to implement within a short timeframe with minimal cost [237]. Other popular models include TNBS–induced colitis, oxazolone-induced colitis, cell transfer models (e.g., adoptive T-cell transfer), and IL-10 knockout [238]. There are many in vitro screening assays to assess the anti-inflammatory effects of various natural products, including cyclooxygenase-2 (COX-2) inhibitory effect assay, bacteria-derived lipopolysaccharide [239]-induced inflammation [239] in human cells (macrophages, and peripheral blood mononuclear cells-PBMCs), myeloperoxidase (MPO) activity assay and inducible nitric oxide synthase (iNOS) inhibitory activity.

### 5.2. Anti-Inflammatory Activities of Plant Extracts and Compounds

Plants produce secondary metabolites, including alkaloids, flavonoids, and phenolics phytochemicals, which can possess anti-inflammatory properties. According to reviews by Ahmad et al. [240], Azab et al. [241], Peng et al. [242], and Salaritabar et al. [243], there is a need for more studies to analyze the efficacy of plant-derived materials/compounds using chronic IBD models and clinical trials. Some natural compounds that have demonstrated anti-inflammatory activities are catechins from *Camellia sinensis* [244], berberine [245] and berberrubine from *Berberis vulgaris* [246], resveratrol from *Polygonum cuspidatum* [247], 14-O-acetylneoline from *Aconitum laciniatum* [248], Bromelain from *Ananas comosus* [249], capnoidine from *Corydalis dubia* [250], zedoarondiol from *Curcuma heyneana* [251], and other pigmented vegetables and fruits. Although numerous pure natural compounds show a range of biological activities, including anti-inflammatory activity, and some are toxic to human cells. While curcumin from *C. longa* is safe after six human trials using concentrations as high as 8000 mg/day for 3 months [252], nevertheless, converting new drug leads from natural sources into effective and safe therapeutic drugs for human consumption may be still a complicated and challenging task. Some promising plant-derived compounds that demonstrate anti-inflammatory activity in various colitis animal models are presented in Table 2 and Table 3.

### 5.3. Fungal-Derived Anti-Inflammatory Drug Leads

To date, more than 133 small molecules were isolated and identified from marine-derived fungi, of which 50 have been tested and showed various anti-inflammatory activities [275]. Small molecules preussins derived from *Aspergillus flocculosus* 16D-1 (a fungus isolated from the marine sponge *Phakellia fusca*) showed potent anti-inflammatory activity (IL-6 inhibitory effects in LPS-induced human monocytic leukemia cell line, THP1) [276]. Graphostromanes F and Khusinol B isolated from *Graphostroma* sp showed inhibition of LPS-induced nitric oxide (NO) production in RAW264.7 macrophages [277,278]. Another small molecule mangicol (A isolated and identified from a marine fungal isolate *Fusarium heterosporum*) also showed anti-inflammatory activity in the phorbol myristate acetate-induced mouse ear edema model [279]. Asperflavin produced by the marine-derived fungus, *Eurotium amstelodami* suppresses iNOS, and pro-inflammatory cytokine production with minimal cytotoxicity [280]. Thus, looking at the great diversity of molecules and their anti-inflammatory activities, natural products, particularly small molecules produced by fungi and related organisms, can offer great potential in developing novel therapeutic drugs against IBD with minimal side effects. Secondary metabolites produced by fungi are also drug leads for many immunosuppressants used in related inflammatory disorders such as mycophenolic acid (produced by *Penicillium* spp.) [281], cyclosporin (produced by *Tolipocladium inflatum*) [282], and fingolimod (Gilenya^TM^) derived from myriocin (produced by *Isaria sinclairii*) [283]. Many such molecules and metabolites derived from various fungi and macrofungi (mushrooms) with potent anti-inflammatory properties are reviewed elsewhere [284,285].

### 5.4. Helminth-Derived Anti-Inflammatories

Studying parasitic helminths and metabolites produced by them at different stages of their life cycles could help to reveal completely novel approaches to suppressing inflammation. Indeed, there is already a substantial body of literature that describes the immunoregulatory properties of these parasites ranging from clinical trials with experimental human helminth infections to animal studies with isolated or synthesized single molecules. Helminths are capable of secreting a vast array of immunoregulatory molecules, including proteins, glycans, lipids, nucleic acids, and small molecule metabolites. For comprehensive reviews on this topic, see [286,287]. Crude excretory/secretory (ES) products or rude soluble extracts (which contain many distinct groups of metabolites) from helminths protect against colitis in mice. For example, the tapeworm *Hymenolepis diminuta* in dinitrobenzene sulfonic acid (DNBS)-induced colitis [288], the roundworm nematode *Trichinella spiralis* in TNBS-induced colitis [289], and the platyhelminth blood fluke *S. mansoni* in adoptive T cell transfer colitis [290]. Wangchuk et al. [291] showed that ES metabolites from the hookworm *Ancyclostoma caninum* could suppress colitis in mouse models by inhibiting the production of inflammatory cytokines. Additionally, Wangchuk et al. [292] identified 54 small molecules from the ES products of the gastrointestinal nematodes *Trichuris muris* and *Nippostrongylus brasiliensis,* of which 17 small molecules possess potent pharmacological activities in the literature. They also reported 49 metabolites from dog tapeworm *Dipylidium caninum,* out of which 12 were bioactive [293]. These studies highlight the untapped metabolomes of parasitic helminths from a therapeutics discovery angle and warrant further attention in the future.

### 5.5. Anti-Inflammatory Peptides

Peptide-based therapeutics are gaining more attention in medical research since several small peptides are proven to have efficacy in experimental colitis in mice [294,295,296,297]. However, delivery of peptides is challenging due to their unstable nature and rapid clearance from circulation. However, when an annexin A1-derived tripeptide MC-12 was grafted within the scaffold of the cyclic peptide sunflower trypsin inhibitor 1 (SFTI-1, isolated from *Helianthus annuus* seeds), it ameliorated acute colitis in mice and displayed enhanced stability [298]. Similarly, grafting this tripeptide into the disulfide-rich peptide linaclotide also improved the stability and bioactivity of the tripeptide [299]. These examples indicate that peptides possess scope for clinical development in IBD if there are promising scaffold peptides like SFTI-1 and linaclotide that can encapsulate and stabilize the bioactive sequence within the peptides of interest.

## 6. Conclusions

IBD continues to cause substantial morbidity and massive productivity loss globally. A single mechanism responsible for IBD is difficult to determine due to the complex interplay of multiple factors, including the host’s genetic predisposition and environmental factors. The relapsing nature of IBD demands repeated treatment, implicating a substantial financial burden to individual patients and the healthcare system, especially in developing nations. The development of SMDs (such as tofacitinib and 5-ASA derivatives mesalamine) and new biologics (integrin antagonist, Entyvio) has revolutionized the treatment of IBD. With the introduction of biologics such as anti-TNF drugs, there is a decreased rate of early surgery in IBD patients. However, there are still challenges with the existing IBD treatment regimens, and new therapeutic modalities are urgently needed. Many small molecules or compounds derived from natural products based on traditional use knowledge have shown promising anti-inflammatory activities in various experimental murine colitis models with few side effects and have the potential to be next-generation drugs. Some small molecules derived from plants such as berberine, curcumin, epigallocatechin-3-gallate, and triptolide are already in clinical trials. An anti-mycobacterium drug (Oral capsule RHB-104), which is repurposed for treating IBD and currently in phase III trial, is another good example of potential IBD drug in the pipeline. Designing a new and non-invasive IBD drug delivery system targeting specific receptors in the colon could primarily help to mitigate the current treatment challenges associated with many side effects. Ethnopharmacology knowledge-guided drug discovery, mainly focusing on small molecules and peptides of medicinal plants, holds promise for the development of safe and novel therapeutics for IBD.

## Figures and Tables

**Table 1:** Current drugs for inflammatory bowel diseases.

Drug Name	Compound Class	Trade Name(S) ^a^	FDA Approved Year	Drug Class	ROA	Half Life ^a^	Target	Mechanism of Action	Major Side Effects	Ref.
**(I) Small Molecule Drugs**										
Azathioprine	Imidazolyl derivative of mercaptopurine	Azasan, Imuran	1999	Immunosuppressant	Oral	~2 h	CD	Metabolism of azathioprine yields 6-thioguanine (6-TGn) nucleotide that inhibits lymphocyte proliferation. 6-TGN is also thought to play role in signalling lymphocyte apoptosis by inhibiting Rac1 activation in T cells.	Nausea, vomiting, leukopenia, and increased susceptibility to infection.	[141]
Mesalamine	5-aminosalicylic acid derivatives	Apriso, Asacol HD, Canasa, Delzicol, Lialda, Pentasa, Rowasa, SfRowasa	1997	5-Aminosalicylic acid derivative	Oral	Variable; ~ 25 h (range: 2−296 hrs)	UC, CD	Inhibits the NF-Kβ pathway, intestinal epithelial cell injury apoptosis.	Dizziness, rhinitis, sinusitis, nasopharyngitis, back pain, abdominal pain, skin rash, eructation, constipation.	[142,143,144,145]
Tofacitinib	Small molecule derived from n-acylpiperidines	Xeljanz, Xeljanz XR	2012	JAK-inhibitor	Oral	~3−6 h	UC	Inhibits JAK family of proteins (JAK-1, 2, 3 & TYK2), while in UC, it is via inhibition of JAK-1 subsequently downregulate IL-6 and IFN-γ.	Nasopharyngitis, headache, skin rash, diarrhoea, herpes zoster infection, upper respiratory tract infection, increased creatine phosphate.	[146]
**(II) Biologics**										
Budesonide	Epimeric mixture of a non-halogenated glucocorticoid, 16 alpha, 17 alpha-(22R,S)-propylmethylenedioxypregna-1,4-diene-11 beta, 21-diol-3,20-dione.	Pulmicort, Pulmicort Flehaler	2013 for UC 2001 for CD,	Corticosteroids	Oral	2.3 h (children) to 3.6 h (adults).	UC, CD		Respiratory infection, rhinitis, nasopharyngitis, dyspepsia, gastroenteritis, microbial infection, otic infection, and cough.	[142,147,148,149]
Infliximab	Anti-TNF-α monoclonal antibody	Inflectra, Remicade, Renflexis	1998	Cytokines/growth factors	IV	7 to 12 days	CD	Binds to TNF-α, thereby interfering with endogenous TNF-α activity.	Headache, abdominal pain, nausea, anaemia, antibody development, infection, upper respiratory infection, sinusitis, cough, pharyngitis.	[150,151,152]
Adalimumab	Anti-TNF-α monoclonal antibody	Humira, Humira Pediatric Crohn’s Start, Humira Pen,	2002	Cytokines/growth factors	Sub-Q	~2 weeks	UC, CD	Binds to TNF-α and prevent from binding its receptor and inhibit subsequent inflammatory responses.	Headache, skin rash, upper respiratory tract infection, sinusitis, antibody development.	[153,154]
Natalizumab	Humanized IgG4k monoclonal antibody produced in murine myeloma cells.	Tysabri	2004	Adhesion molecules/ chemokines	IV	3−17 days	CD	Blocks integrin (α4 subunit) association with vascular receptors, limiting adhesion and transmigration of leukocytes.	Headache, fatigue, depression, skin rash, nausea, gastroenteritis, abdominal distress, urinary tract infection, influenza, arthralgia, limb pain, back pain, upper respiratory tract infection, flu-like symptoms, peripheral edema, chest discomfort, dermatitis, menstrual disease, diarrhoea, tooth infection, dyspepsia, vaginal infection, urinary tract infection, antibody development, muscle cramp, cough, sinusitis, tonsillitis, and microbial infections.	[155,156]
Certolizumab pegol (CZP)	a recombinant humanized Fab′ fragment of a monoclonal antibody	Cimzia, Cimzia 200mg, Cimzia 200 mg/mL, Cimzia-200	2008	Cytokines/growth factors/ immunosuppressant	Sub-Q	14 days	CD	Selectively neutralizes TNF-α.	Upper respiratory infection, urinary tract infection, arthralgia, rash	[142,157,158]
Golimumab	From genetically engineered mice with human anti-TNF antibody	Simponi, Simponi Aria	2013	Biologic agent, TNF blocking agent	IV	2 weeks	UC	Inhibits TNF-α activity by binding to its receptor.	Respiratory infections (nasopharyngitis), decreased neutrophils, and microbial infections.	[142,159,160,161]
Vedolizumab	Monoclonal antibody	Entyvio	2014	Biologic agent	IV	25 days	UC, CD	Integrin antagonist; and inhibits gut specific α4β7 integrin LPAM 1.	Upper respiratory tract infection, nasopharyngitis, headache, nausea, fatigue, cough, fever, and antibody development.	[142,162]
Ustekinumab	Human immunoglobulin (Ig) G1 kappa monoclonal antibody.	Stelara	2016 for CD; 2019 for UC	Cytokines/growth factor	Sub-Q	~19 days	UC, CD	Binds to, and interferes with the proinflammatory cytokines, IL-12 and IL-23. Ustekinumab also interferes with the expression of monocyte chemotactic protein-1 (MCP-1), TNF-α, interferon-inducible protein-10, and IL-8.	Antibody development, nasopharyngitis, headache, vaginal mycosis, vulvovaginal candidiasis, erythema at injection site, and bronchitis.	[163,164]

^a^ All information on trade name, drug class and half-life are referred from http://www.drugs.com, and http://www.drugbank.ca. FDA, Food and Drug Administration; ROA, Route of Administration; CD, Crohn’s disease; UC, ulcerative colitis; NF-Kβ, nuclear factor kappa β; JAK, Janus kinase; TYK2, Tyrosine kinase-2; IL, Interleukin; IFN-γ, Interferon gamma; IV, Intravenous; TNF-α, Tumour Necrosis Factor alpha; LPAM, lymphocyte Peyer’s patch adhesion molecule.

**Table 2:** Plant-derived compounds in various phases of clinical trials for Inflammatory Bowel Diseases ^a^.

Name	Plant	Disease/Condition	Target/Objective	Clinical Phase	Location(s)/Developer
Berberine	*Coptis chinensis* Franch	UC	Assess the safety of berberine (berberine chloride) for UC patients in clinical remission while receiving maintenance therapy with mesalamine.	Phase I	Northwestern University Chicago, Illinois, United States. Fourth Military Medical University Xi’an, Shaanxi, China.
Epigallocatechin-3-gallate	*C. sinensis* L.	Mild to moderately active UC	Determine the Safety of an oral dose of green tea extract (Polyphenon E^®^) as a preliminary evidence to support its efficacy in UC.	Phase II	University of Louisville Clinical Research Center Louisville, Kentucky, United States.
Triptolide	*Tripterygium wilfordii* Hook. F.	CD	Assess the effect and safety of Tripterygium Glycosides in the treatment of CD for induction remission and compare the therapeutic effect with patients who received mesalamine.	Phase II Phase III	General Surgery Institute, Jinling Hospital Nanjing, Jiangsu, China.
Curcumin (1,7-Bis(4-hydroxy-3-methoxyphenyl)-1,6-heptadiene-3,5-dione)	*C. longa* L.	Both UC and CD	Determine the tolerability of curcumin in pediatric IBD patients.	Phase I	Seattle Children’s Hospital Seattle, Washington, United States.
		CD	Study the effect of curcumin combined with thiopurines in the prevention of post-operative recurrence of CD.	Phase III	University Hospital of Clermont-Ferrand (CHU), Clermont-Ferrand, France.
		UC	Evaluate the efficacy of combined therapy of curcumin + 5ASA versus 5ASA alone on mild to moderate UC patients.	Phase III	Sheba Medical Center Ramat Gan, Israel.

^a^ All resources were obtained from www.clinicaltrials.gov. UC, ulcerative colitis; CD, Crohn’s disease.

**Table 3:** Natural products showing protective functions in various animal colitis models/cell lines.

Source	Isolated Compounds	Animal Models/Cell Lines	The Main Effect on Inflammation	Ref
*A. laciniatum*	14-O-acetylneoline	TNBS-induced mice.	Protects colonic inflammation and reduces colonic IFN-γ mRNA levels.	[248]
*Andrographis paniculata*	Ethanolic extract	Pelvic inflammatory disease induced Sprague Dawley rats.	Inhibits NF-kB signal pathway.	[253]
*Ananas comosus*	Bromelain	LPS-induced human intestinal adenocarcinoma cell line (HT29 cells); DSS-induced colitis mice.	Reduces mRNA expression of proinflammatory cytokines IL-8 and TNF-α in LPS challenged HT29 cells; reduces inflammation in DSS-induced colitis mice.	[249]
*Amphipterygium adstringens*	Alcoholic extract	DSS-induced mice BALB/c mice.	Significant reduction in levels of inflammatory cytokines TNF-α, IFN-γ, and IL-1β.	[254]
*Aralia continentalis*	Kaurenoic acid (ent-kaur-16-en-19-oic acid)	LPS-induced RAW264.7 macrophages.	Significant reduction of the diameter in carrageenan-induced paw edema mice model; Suppression of the COX-2 activity.	[255]
*Basella rubra*	Methanolic extract	Oxazolone-induced rats.	Enhances recovery from colon inflammation.	[256]
*Cinnamomum verum*	Trans-cinnamaldehyde and p-cymene	THP1 monocyte-macrophage cell line TIB-202 (ATCC).	Significant reduction of the LPS-dependent IL-8 secretion in THP1 monocytes.	[257]
*C. dubia*	Capnoidine	TNBS-induced colitis mice.	Reduces colon pathology and inflammation; reduces p-IκB-α (Ser32) and p-NF-κB p65 (Ser536) levels.	[250]
*C. heyneana*	Zedoarondiol	LPS-induced macrophage cell.	Dose dependent inhibition of LPS-stimulated NO, prostaglandin E2, IL-1β, IL-6, and TNF-α in RAW 264.7 macrophage and mouse peritoneal macrophage cells.	[251]
*Cryptostegia grandiflora*	Leaf ethanol extract	12-O -tetradecanoyl-phorbol-13-acetate (TPA) treated mice.	Reduces inflammation and MPO in ear tissue; reduces edema and leukocyte infiltration.	[258]
*Euphorbia tirucalli*	Euphol	DSS- and TNBS-induced mice.	Inhibits the levels and expression of IL-1β, CXCL1/KC, MCP-1, MIP-2, TNF-α and IL-6 in colonic tissue; reduces the expression of NOS2, VEGF, and Ki67 in colonic tissue.	[259]
*Evodia rutaecarpa*	Evodiamine, Rutaecarpine	LPS induced-RAW 267.7 cell.	Inhibits PGE2 production.	[260]
*Evodia fructus*	Dehydroevodiamine	LPS-induced RAW 264.7 macrophages cells.	Produces mark PGE2 and COX-2 inhibition via inhibiting the NF-κB activity.	[260]
*Fissistigma oldhamii*	7′-(3′,4′-dihydroxyphenyl)-n-[(4-methoxyphenyl) ethyl] propenamide (Z23)	LPS-induced RAW 264.7 macrophages.	Exhibits mark PGE2 inhibition via suppressing the COX-2 expression.	[261]
*Ganoderma lucidum*	DMSO extract	LPS stimulation on cancer cells.	Reduces the levels of IL-6, IL-8, MMP-2, MMP-9 in breast cancer cells.	[262]
*Ipomoea asarifolia*	Aqueous extract	Dinitrobenzene sulfonic acid (DNBS)-induced colitis in mice.	Reduces MPO activity; down-regulates the gene expression of JNK1, NF-kβ-p65, and STAT3; decreases the level of TNF-α, and IL-1β, and increases IL-10.	[263]
*Millettia pulchra*	Ethanol extract and isolated compound lanceolatin B	Xylene-induced ear edema mice.	Reduces pain; inhibits NO synthesis.	[264]
*Panax quinquefolius*	Ginsenosides	azoxymethane [265]/ DSS mouse model.	Inhibits inflammatory cytokines and restores microbiome inhibited by AOM/DSS.	[266]
*P. quinquefolius*	Aqueous extract	Acetic acid-induced UC rats.	Heals colon tissues.	[267]
	Gallic acid	TNBS-induced UC in rats.	Reduces MPO activity.	[268]
*Penicillium paxillin*	Pyrenocine A	LPS-induced RAW 264.7 cell line.	Inhibits nitrite production and the synthesis of proinflammatory cytokines and PGE2.	[269]
*Pistacia atlantica*	Methanolic leaf extract	Carrageenan-induced mice.	Reduces hind paw edema.	[270]
*Serpylli herba*	Aqueous extract	TNBS-induced rat colitis.	Reduces the expression of proinflammatory cytokines (TNF-α, IL-1β, IFN-γ, IL-6, and IL-17), the chemokine (MCP-1), and the adhesion molecule (ICAM-1).	[271]
*Stephania tetrandra*	Methanolic extract of roots	Silica stimulated human monocytes.	Reduces the level of IL-6.	[272]
*Sida cordifolia*	5′-Hydroxymethyl-1′- (1,2,3,9-tetrahydro-pyrrolo [2,1-b] quinazolin-1-yl)-heptan-1-one)	Carrageenan-induced rat paw edema model.	Inhibits rat paw edema.	[273]
*Styrax japonica*	Styraxosides A	RAW 264.7 Cells.	Inhibits protein the expression levels of nitric oxide synthase (NOS) and cyclooxygenase-2 (COX-2); mRNA expression levels of NOS and COX-2, TNF-α, IL-1β; inhibits DNA binding activity of NF-kB pathway.	[274]

IFN-γ, interferon gamma; LPS, lipopolysaccharide; DSS, dextran sulfate sodium; BALB/c, Bagg Albino C; RAW, Ralph And William’s cell line; COX-2, cyclooxygenase-2; THP1, human monocytic leukemia cell line; NO, nitric oxide; MPO, myeloperoxidase; PGE2, prostaglandin E_2_; DMSO, dimethyl sulfoxide; MMP, matrix metalloproteinase.

## References

[1] Mulder D.J., Noble A.J., Justinich C.J., Duffin J.M. (2014). A tale of two diseases: The history of inflammatory bowel disease. J. Crohn’s Colitis.

[2] Aufses A.H. (2001). The history of Crohn’s Disease. Surg. Clin. N. Am..

[3] Alatab S., Sepanlou S.G., Ikuta K., Vahedi H., Bisignano C., Safiri S., Sadeghi A., Nixon M.R., Abdoli A., Abolhassani H. (2019). The global, regional, and national burden of inflammatory bowel disease in 195 countries and territories, 1990–2017: A systematic analysis for the Global Burden of Disease Study 2017. Lancet Gastroenterol. Hepatol.

[4] Aniwan S., Tremaine W.J., Raffals L.E., Kane S.V., Loftus E.V. (2018). Antibiotic Use and New-Onset Inflammatory Bowel Disease in Olmsted County, Minnesota: A Population-Based Case-Control Study. J. Crohn’s Colitis.

[5] Ng S.C., Tang W., Ching J.Y., Wong M., Chow C.M., Hui A.J., Wong T.C., Leung V.K., Tsang S.W., Yu H.H. (2013). Incidence and phenotype of inflammatory bowel disease based on results from the Asia-pacific Crohn’s and colitis epidemiology study. Gastroenterology.

[6] Kaplan G.G., Ng S.C. (2016). Globalisation of inflammatory bowel disease: Perspectives from the evolution of inflammatory bowel disease in the UK and China. Lancet Gastroenterol. Hepatol..

[7] Inflammatory Bowel Disease National Action Plan 2019. https://www.crohnsandcolitis.com.au/site/wp-content/uploads/National-Action-Plan-FINAL-08-03-2019.pdf.

[8] Yang Y., Owyang C., Wu G.D. (2016). East Meets West: The Increasing Incidence of Inflammatory Bowel Disease in Asia as a Paradigm for Environmental Effects on the Pathogenesis of Immune-Mediated Disease. Gastroenterology.

[9] Asakura K., Nishiwaki Y., Inoue N., Hibi T., Watanabe M., Takebayashi T. (2009). Prevalence of ulcerative colitis and Crohn’s disease in Japan. J. Gastroenterol..

[10] Kwak M.S., Cha J.M., Lee H.H., Choi Y.S., Seo S.I., Ko K.J., Park D.I., Kim S.H., Kim T.J. (2019). Emerging trends of inflammatory bowel disease in South Korea: A nationwide population-based study. J. Gastroenterol. Hepatol..

[11] Yen H.H., Weng M.T., Tung C.C., Wang Y.T., Chang Y.T., Chang C.H., Shieh M.J., Wong J.M., Wei S.C. (2019). Epidemiological trend in inflammatory bowel disease in Taiwan from 2001 to 2015: A nationwide populationbased study. Intest. Res..

[12] Ferguson A., Sedgwick D.M., Drummond J. (1994). Morbidity of juvenile onset inflammatory bowel disease: Effects on education and employment in early adult life. Gut.

[13] Kappelman M.D., Rifas-Shiman S.L., Porter C.Q., Ollendorf D.A., Sandler R.S., Galanko J.A., Finkelstein J.A. (2008). Direct health care costs of Crohn’s disease and ulcerative colitis in US children and adults. Gastroenterology.

[14] Longobardi T., Jacobs P., Bernstein C.N. (2003). Work losses related to inflammatory bowel disease in the United States results from the National Health Interview Survey. Am. J. Gastroenterol..

[15] Kaplan G.G. (2015). The global burden of IBD: From 2015 to 2025. Nat. Rev. Gastroenterol. Hepatol..

[16] M’Koma A.E. (2013). Inflammatory bowel disease: An expanding global health problem. Clin. Med. Insights Gastroenterol..

[17] Park K.T., Ehrlich O.G., Allen J.I., Meadows P., Szigethy E.M., Henrichsen K., Kim S.C., Lawton R.C., Murphy S.M., Regueiro M. (2020). The Cost of Inflammatory Bowel Disease: An Initiative From the Crohn’s & Colitis Foundation. Inflamm. Bowel Dis..

[18] Improving Inflammatory Bowel Disease Care across Australia. https://www.crohnsandcolitis.com.au/site/wp-content/uploads/PwC-report-2013.pdf.

[19] Jeong D.Y., Kim S., Son M.J., Son C.Y., Kim J.Y., Kronbichler A., Lee K.H., Shin J.I. (2019). Induction and maintenance treatment of inflammatory bowel disease: A comprehensive review. Autoimmun. Rev..

[20] Neurath M.F. (2017). Current and emerging therapeutic targets for IBD. Nat. Rev. Gastroenterol. Hepatol..

[21] Ananthakrishnan A.N. (2015). Epidemiology and risk factors for IBD. Nat. Rev. Gastroenterol. Hepatol..

[22] Ruel J., Ruane D., Mehandru S., Gower-Rousseau C., Colombel J.F. (2014). IBD across the age spectrum: Is it the same disease?. Nat. Rev. Gastroenterol. Hepatol..

[23] Zhang Y.Z., Li Y.Y. (2014). Inflammatory bowel disease: Pathogenesis. World J. Gastroenterol..

[24] Baumgart D.C., Sandborn W.J. (2007). Inflammatory bowel disease: Clinical aspects and established and evolving therapies. Lancet.

[25] Guindi M., Riddell R.H. (2004). Indeterminate colitis. J. Clin. Pathol..

[26] Tremaine W.J. (2011). Diagnosis and Treatment of Intermediate Colitis. Gastroenterol. Hepatol..

[27] Teixeira F.V., Hosne R.S., Sobrado C.W. (2015). Management of ulcerative colitis: A clinical update. J. Coloproctol..

[28] https://www.crohnsandcolitis.org.uk/about-crohns-and-colitis/publications/ulcerative-colitis.

[29] Schroeder K.W., Tremaine W.J., Ilstrup D.M. (1987). Coated Oral 5-Aminosalicylic Acid Therapy for Mildly to Moderately Active Ulcerative Colitis. N. Engl. J. Med..

[30] Dignass A., Eliakim R., Magro F., Maaser C., Chowers Y., Geboes K., Mantzaris G., Reinisch W., Colombel J.F., Vermeire S. (2012). Second European evidence-based consensus on the diagnosis and management of ulcerative colitis part 1: Definitions and diagnosis. J. Crohn’s Colitis.

[31] Jess T., Rungoe C., Peyrin-Biroulet L. (2012). Risk of colorectal cancer in patients with ulcerative colitis: A meta-analysis of population-based cohort studies. Clin. Gastroenterol. Hepatol..

[32] Frank D.N., St Amand A.L., Feldman R.A., Boedeker E.C., Harpaz N., Pace N.R. (2007). Molecular-phylogenetic characterization of microbial community imbalances in human inflammatory bowel diseases. Proc. Natl. Acad. Sci. USA.

[33] Roediger W.E., Moore J., Babidge W. (1997). Colonic Sulfite in Pathogenesis and Treatment of Ulcerative Colitis. Dig. Dis. Sci..

[34] Bamias G., Nyce M.R., Sarah A., Cominelli F. (2005). New Concepts in the Pathophysiology of Inflammatory Bowel Disease. Ann. Intern. Med..

[35] Fuss I.J., Neurath M., Boirivant M., Klein J.S., De La Motte C., Strong S.A., Fiocchi C., Strober W. (1996). Disparate CD4+ Lamina Propria (LP)Lymphokine Secretion Profiles in Inflammatory Bowel Disease Crohn’s Disease LP Cells Manifest Increased Secretion of IFN-7, Whereas UlcerativeColitisLPCellsManifestIncreasedSecretion ofIL-5. J. Immunol..

[36] Strober W., Fuss I.J. (2011). Proinflammatory cytokines in the pathogenesis of inflammatory bowel diseases. Gastroenterology.

[37] Heller F., Peter F., Bojarski C., Richter J., Christ M., Hillenbrand B., Mankertz J., Gitter A.H., Bürgel N., Fromm M. (2005). Interleukin-13 Is the Key Effector Th2 Cytokine in Ulcerative Colitis That Affects Epithelial Tight Junctions, Apoptosis, and Cell Restitution. Gastroenterology.

[38] Heller F., Fromm A., Gitter A.H., Mankertz J., Schulzke J.D. (2008). Epithelial apoptosis is a prominent feature of the epithelial barrier disturbance in intestinal inflammation: Effect of pro-inflammatory interleukin-13 on epithelial cell function. Mucosal. Immunol..

[39] Ungaro R., Mehandru S., Allen P.B., Peyrin-Biroulet L., Colombel J.-F. (2017). Ulcerative colitis. Lancet.

[40] Raphael I., Nalawade S., Eagar T.N., Forsthuber T.G. (2015). T cell subsets and their signature cytokines in autoimmune and inflammatory diseases. Cytokine.

[41] Nalleweg N., Chiriac M.T., Podstawa E., Lehmann C., Rau T.T., Atreya R., Krauss E., Hundorfean G., Fichtner-Feigl S., Hartmann A. (2015). IL-9 and its receptor are predominantly involved in the pathogenesis of UC. Gut.

[42] Hokari R., Kato S., Matsuzaki K., Iwai A., Kawaguchi A., Nagao S., Miyahara T., Itoh K., Sekizuka E., Nagata H. (2001). Involvement of mucosal addressin cell adhesion molecule-1 (MAdCAM-1) in the pathogenesis of granulomatous colitis in rats. Clin. Exp. Immunol..

[43] Arihiro S., Ohtani H., Suzuki M., Murata M., Ejima C., Oki M., Kinouchi Y., Fukushima K., Sasaki I., Nakamura S. (2002). Differential expression of mucosal addressin cell adhesion molecule-1 (MAdCAM-1) in ulcerative colitis and Crohn’s disease. Pathol. Int..

[44] Jussila A., Virta L.J., Pukkala E., Farkkila M.A. (2014). Mortality and causes of death in patients with inflammatory bowel disease: A nationwide register study in Finland. J. Crohn’s Colitis.

[45] Winther K.V., Jess T., Langholz E., Munkholm P., Binder V. (2003). Survival and Cause-Specific Mortality in Ulcerative Colitis: Follow-up of a Population-Based Cohort in Copenhagen County. Gastroenterology.

[46] Wilkins T., Jarvis K., Patel J. (2011). Diagnosis and Management of Crohn’s Disease. Am. Fam. Physician.

[47] Van Rheenen P.F., Van de Vijver E., Fidler V. (2010). Faecal calprotectin for screening of patients with suspected inflammatory bowel disease: Diagnostic meta-analysis. BMJ.

[48] Røseth A.G., Aadland E., Jahnsen J., Raknerud N. (1997). Assessment of disease activity in ulcerative colitis by faecal calprotein, a novel granulocyte marker protein. Digestion.

[49] D’Haens G., Ferrante M., Vermeire S., Baert F., Noman M., Moortgat L., Geens P., Iwens D., Aerden I., Van Assche G. (2012). Fecal calprotectin is a surrogate marker for endoscopic lesions in inflammatory bowel disease. Inflamm. Bowel Dis..

[50] Li F., Ma J., Geng S., Wang J., Liu J., Zhang J., Sheng X. (2015). Fecal calprotectin concentrations in healthy children aged 1-18 months. PLoS ONE.

[51] Mumolo M.G., Bertani L., Ceccarelli L., Laino G., Di Fluri G., Albano E., Tapete G., Costa F. (2018). From bench to bedside: Fecal calprotectin in inflammatory bowel diseases clinical setting. World J. Gastroenterol..

[52] Kolls J.K., Linden A. (2004). Interleukin-17 family members and inflammation. Immunity.

[53] Matsuoka K., Inoue N., Sato T., Okamoto S., Hisamatsu T., Kishi Y., Sakuraba A., Hitotsumatsu O., Ogata H., Koganei K. (2004). T-bet upregulation and subsequent interleukin 12 stimulation are essential for induction of Th1 mediated immunopathology in Crohn’s disease. Gut.

[54] Sartor R.B. (2006). Mechanisms of disease: Pathogenesis of Crohn’s disease and ulcerative colitis. Nat. Clin. Pract. Gastroenterol. Hepatol..

[55] Strachan D.P. (1989). Hay fever, hygiene, and household size. BMJ.

[56] Bach J.F. (2018). The hygiene hypothesis in autoimmunity: The role of pathogens and commensals. Nat. Rev. Immunol..

[57] Greenwood B.M., Herrick E.M., Voller A. (1970). Suppression of autoimmune disease in NZB and (NZB × NZW) F1 hybrid mice by infection with malaria. Nature.

[58] Greenwood B.M., Herrick E.M., Voller A. (1970). Can parasitic infection suppress autoimmune disease?. Proc. R. Soc. Med..

[59] Sewell D.L., Reinke E.K., Hogan L.H., Sandor M., Fabry Z. (2002). Immunoregulation of CNS autoimmunity by helminth and mycobacterial infections. Immunol. Lett..

[60] Rook G.A., Brunet L.R. (2005). Microbes, immunoregulation, and the gut. Gut.

[61] Feeney M.A., Murphy F., Clegg A.J., Trebble T.M., Sharer N.M., Snook J.A. (2002). A case–control study of childhood environmental risk factors for the development of inflammatory bowel disease. Eur. J. Gastroenterol. Hepatol..

[62] Jostins L., Ripke S., Weersma R.K., Duerr R.H., McGovern D.P., Hui K.Y., Lee J.C., Schumm L.P., Sharma Y., Anderson C.A. (2012). Host-microbe interactions have shaped the genetic architecture of inflammatory bowel disease. Nature.

[63] Khor B., Gardet A., Xavier R.J. (2011). Genetics and pathogenesis of inflammatory bowel disease. Nature.

[64] Tysk C., Lindberg E., Järnerot G., Floderus-Myrhed B. (1988). Ulcerative colitis and Crohn’s disease in an unselected population of monozygotic and dizygotic twins. A study of heritability and the influence of smoking. Gut.

[65] Thompson N.P., Driscoll R., Pounder R.E., Wakefield A.J. (1996). Genetics versus environment in inflammatory bowel disease: Results of a British twin study. BMJ.

[66] Halfvarson J., Bodin L., Tysk C., Lindberg E., Järnerot G. (2003). Inflammatory bowel disease in a Swedish twin cohort: A long-term follow-up of concordance and clinical characteristics. Gastroenterology.

[67] Halme L., Paavola-Sakki P., Turunen U., Lappalainen M., Färkkilä M., Kontula K. (2006). Family and twin studies in inflammatory bowel disease. World J. Gastroenterol..

[68] Khalili H., Huang E.S., Ananthakrishnan A.N., Higuchi L., Richter J.M., Fuchs C.S., Chan A.T. (2012). Geographical variation and incidence of inflammatory bowel disease among US women. Gut.

[69] Maeda A., Beissert S., Schwarz T., Schwarz A. (2008). Phenotypic and functional characterization of ultraviolet radiation-induced regulatory T cells. J. Immunol..

[70] Maeda S.S., Saraiva G.L., Hayashi L.F., Cendoroglo M.S., Ramos L.R., Correa Mde P., Henrique de Mesquita C., Lazaretti-Castro M. (2013). Seasonal variation in the serum 25-hydroxyvitamin D levels of young and elderly active and inactive adults in Sao Paulo, Brazil: The Sao PAulo Vitamin D Evaluation Study (SPADES). Dermatoendocrinol.

[71] Froicu M., Weaver V., Wynn T.A., McDowell M.A., Welsh J.E., Cantorna M.T. (2003). A crucial role for the vitamin D receptor in experimental inflammatory bowel diseases. Mol. Endocrinol..

[72] Ng S.C., Tang W., Leong R.W., Chen M., Ko Y., Studd C., Niewiadomski O., Bell S., Kamm M.A., de Silva H.J. (2015). Environmental risk factors in inflammatory bowel disease: A population-based case-control study in Asia-Pacific. Gut.

[73] Pinsk V., Lemberg D.A., Grewal K., Barker C.C., Schreiber R.A., Jacobson K. (2007). Inflammatory bowel disease in the South Asian pediatric population of British Columbia. Am. J. Gastroenterol..

[74] Probert C.S., Jayanthi V., Pinder D., Wicks A.C., Mayberry J.F. (1992). Epidemiological study of ulcerative proctocolitis in Indian migrants and the indigenous population of Leicestershire. Gut.

[75] Benchimol E.I., Mack D.R., Guttmann A., Nguyen G.C., To T., Mojaverian N., Quach P., Manuel D.G. (2015). Inflammatory bowel disease in immigrants to Canada and their children: A population-based cohort study. Am. J. Gastroenterol..

[76] Timm S., Svanes C., Janson C., Sigsgaard T., Johannessen A., Gislason T., Jogi R., Omenaas E., Forsberg B., Toren K. (2014). Place of upbringing in early childhood as related to inflammatory bowel diseases in adulthood: A population-based cohort study in Northern Europe. Eur. J. Epidemiol..

[77] Zoetendal E.G., Puylaert P.G., Ou J., Vipperla K., Brouard F.M., Ruder E.H., Newton K., Carbonero F., Gaskins H.R., de Vos W.M. (2013). Sa1968 Distinct Microbiotas Are Present in Urban and Rural Native South Africans, and in African Americans. Gastroenterology.

[78] Das B., Ghosh T.S., Kedia S., Rampal R., Saxena S., Bag S., Mitra R., Dayal M., Mehta O., Surendranath A. (2018). Analysis of the Gut Microbiome of Rural and Urban Healthy Indians Living in Sea Level and High Altitude Areas. Sci. Rep..

[79] Rakoff-Nahoum S., Paglino J., Eslami-Varzaneh F., Edberg S., Medzhitov R. (2004). Recognition of commensal microflora by toll-like receptors is required for intestinal homeostasis. Cell.

[80] Nagalingam N.A., Lynch S.V. (2012). Role of the microbiota in inflammatory bowel diseases. Inflamm. Bowel Dis..

[81] Eckburg P.B., Bik E.M., Bernstein C.N., Purdom E., Dethlefsen L., Sargent M., Gill S.R., Nelson K.E., Relman D.A. (2005). Diversity of the Human Intestinal Microbial Flora. Science.

[82] Morgan X.C., Tickle T.L., Sokol H., Gevers D., Devaney K.L., Ward D.V., Reyes J.A., Shah S.A., LeLeiko N., Snapper S.B. (2012). Dysfunction of the intestinal microbiome in inflammatory bowel disease and treatment. Genome Biol..

[83] Flint H.J., Bayer E.A., Rincon M.T., Lamed R., White B.A. (2008). Polysaccharide utilization by gut bacteria: Potential for new insights from genomic analysis. Nat. Rev. Microbiol..

[84] Sokol H., Seksik P., Furet J.P., Firmesse O., Nion-Larmurier I., Beaugerie L., Cosnes J., Corthier G., Marteau P., Dore J. (2009). Low counts of Faecalibacterium prausnitzii in colitis microbiota. Inflamm. Bowel Dis..

[85] Martin R., Chain F., Miquel S., Lu J., Gratadoux J.J., Sokol H., Verdu E.F., Bercik P., Bermudez-Humaran L.G., Langella P. (2014). The commensal bacterium Faecalibacterium prausnitzii is protective in DNBS-induced chronic moderate and severe colitis models. Inflamm. Bowel Dis..

[86] David L.A., Maurice C.F., Carmody R.N., Gootenberg D.B., Button J.E., Wolfe B.E., Ling A.V., Devlin A.S., Varma Y., Fischbach M.A. (2014). Diet rapidly and reproducibly alters the human gut microbiome. Nature.

[87] Devkota S., Wang Y., Musch M.W., Leone V., Fehlner-Peach H., Nadimpalli A., Antonopoulos D.A., Jabri B., Chang E.B. (2012). Dietary-fat-induced taurocholic acid promotes pathobiont expansion and colitis in Il10-/- mice. Nature.

[88] Turnbaugh P.J., Ridaura V.K., Faith J.J., Rey F.E., Knight R., Gordon J.I. (2009). The effect of diet on the human gut microbiome: A metagenomic analysis in humanized gnotobiotic mice. Sci. Transl. Med..

[89] Wu G.D., Chen J., Hoffmann C., Bittinger K., Chen Y.Y., Keilbaugh S.A., Bewtra M., Knights D., Walters W.A., Knight R. (2011). Linking long-term dietary patterns with gut microbial enterotypes. Science.

[90] Ananthakrishnan A.N., Khalili H., Konijeti G.G., Higuchi L.M., de Silva P., Korzenik J.R., Fuchs C.S., Willett W.C., Richter J.M., Chan A.T. (2013). A prospective study of long-term intake of dietary fiber and risk of Crohn’s disease and ulcerative colitis. Gastroenterology.

[91] Jick H., Walker A.M. (1983). Cigarettesmokingandulcerativecolitis. N. Engl. J. Med..

[92] Gyde S., Prior P., Dew M.J., Saunders V., Waterhouse J.A., Allan R.N. (1982). Mortality in ulcerative colitis. Gastroenterology.

[93] Vessey M., Jewell D., Smith A., Yeates D., McPherson K. (1986). Chronic inflammatory bowel disease, cigarette smoking, and use of oral contraceptives: Findings in a large cohort study of women of childbearing age. Br. Med. J. (Clin. Res. Ed.).

[94] Pullan R.D., Rhodes J., Ganesh S., Mani V., Morris J.S., Williams G.T., Newcombe R.G., Russel M.A.H., Feyerabend C., Thomas G.A.O. (1994). Transdermal Nicotine for Active Ulcerative Colitis. N. Engl. J. Med..

[95] Uemura R., Fujiwara Y., Iwakura N., Shiba M., Watanabe K., Kamata N., Yamagami H., Tanigawa T., Watanabe T., Tominaga K. (2016). Sleep disturbances in Japanese patients with inflammatory bowel disease and their impact on disease flare. Springerplus.

[96] Ananthakrishnan A.N., Khalili H., Konijeti G.G., Higuchi L.M., de Silva P., Fuchs C.S., Richter J.M., Schernhammer E.S., Chan A.T. (2014). Sleep duration affects risk for ulcerative colitis: A prospective cohort study. Clin. Gastroenterol. Hepatol..

[97] Mikocka-Walus A., Pittet V., Rossel J.B., von Kanel R. (2016). Symptoms of Depression and Anxiety Are Independently Associated With Clinical Recurrence of Inflammatory Bowel Disease. Clin. Gastroenterol. Hepatol..

[98] Mikocka-Walus A., Massuger W., Knowles S.R., Moore G.T., Buckton S., Connell W., Pavli P., Raven L., Andrews J.M. (2019). Psychological distress is highly prevalent in inflammatory bowel disease: A survey of psychological needs and attitudes. JGH Open.

[99] Lerebours E., Gower-Rousseau C., Merle V., Brazier F., Debeugny S., Marti R., Salomez J.L., Hellot M.F., Dupas J.L., Colombel J.F. (2007). Stressful life events as a risk factor for inflammatory bowel disease onset: A population-based case-control study. Am. J. Gastroenterol..

[100] Swanson G.R., Burgess H.J., Keshavarzian A. (2011). Sleep disturbances and inflammatory bowel disease: A potential trigger for disease flare?. Expert Rev. Clin. Immunol..

[101] Ananthakrishnan A.N., Long M.D., Martin C.F., Sandler R.S., Kappelman M.D. (2013). Sleep disturbance and risk of active disease in patients with Crohn’s disease and ulcerative colitis. Clin. Gastroenterol. Hepatol..

[102] Mardini H.E., Kip K.E., Wilson J.W. (2004). Crohn’s Disease: A Two-Year Prospective Study of the Association Between Psychological Distress and Disease Activity. Dig. Dis. Sci..

[103] Levenstein S., Prantera C., Varvo V., Scribano M.L., Andreoli A., Luzi C., Arca M., Berto E., Milite G., Marcheggiano A. (2000). Stress and exacerbation in Ulcerative Colitis: A prospective study of patients enrolled in remission. Am. J. Gastroenterol..

[104] Radford-Smith G.L. (2008). What is the importance of appendectomy in the natural history of IBD?. Inflamm. Bowel Dis..

[105] Mizoguchi A., Mizoguchi E., Chiba C., Bhan A.K. (1996). Role of Appendix in the Development of Inflammatory Bowel Disease in TCR-c~ Mutant Mice. J. Exp. Med..

[106] Mombaerts P.M., Mizoguchi E., Grusby M.J., Glimcher L.H., Bhan A.K., Tonegawa S. (1993). Spontaneous Development of Inflammatory Bowel Disease in T Cell Receptor Mutant Mice. Cell.

[107] Radford-Smith G.L., Edwards J.E., Purdie D.M., Pandeya N., Watson M., Martin N.G., Green A., Newman B., Florin T.H. (2002). Protective role of appendicectomy on onset and severity of ulcerative colitis and Crohn’s disease. Gut.

[108] Noh C.H., Cheung D.Y., Kim T.H., Jun E.J., Lee I.K., Kim J.I., Cho S.H., Park S.H., Han J.Y., Kim J.K. (2010). Remission of ulcerative colitis after appendectomy: A case report. Korean J. Gastroenterol..

[109] Shaw S.Y., Blanchard J.F., Bernstein C.N. (2010). Association between the use of antibiotics in the first year of life and pediatric inflammatory bowel disease. Am. J. Gastroenterol..

[110] Hviid A., Svanstrom H., Frisch M. (2011). Antibiotic use and inflammatory bowel diseases in childhood. Gut.

[111] Shaw S.Y., Blanchard J.F., Bernstein C.N. (2011). Association between the use of antibiotics and new diagnoses of Crohn’s disease and ulcerative colitis. Am. J. Gastroenterol..

[112] Virta L., Auvinen A., Helenius H., Huovinen P., Kolho K.L. (2012). Association of repeated exposure to antibiotics with the development of pediatric Crohn’s disease—A nationwide, register-based finnish case-control study. Am. J. Epidemiol..

[113] Garcia Rodriguez L.A., Ruigomez A., Panes J. (2006). Acute gastroenteritis is followed by an increased risk of inflammatory bowel disease. Gastroenterology.

[114] Kronman M.P., Zaoutis T.E., Haynes K., Feng R., Coffin S.E. (2012). Antibiotic exposure and IBD development among children: A population-based cohort study. Pediatrics.

[115] Chan S.S., Luben R., Bergmann M.M., Boeing H., Olsen A., Tjonneland A., Overvad K., Kaaks R., Kennedy H., Khaw K.T. (2011). Aspirin in the aetiology of Crohn’s disease and ulcerative colitis: A European prospective cohort study. Aliment. Pharmacol. Ther..

[116] Ordás I., Eckmann L., Talamini M., Baumgart D.C., Sandborn W.J. (2012). Ulcerative colitis. Lancet.

[117] Knutson D., Greenberg G., Cronau H. (2003). Management of Crohn’s Disease—A Practical Approach. Am. Fam. Physician.

[118] Faubion W.A.J., Loftus E.V.J., Harmsen W.S., Zinsmeister A.R., Sandborn W.J. (2001). The natural history of corticosteroid therapy for inflammatory bowel disease: A population-based study. Gastroenterology.

[119] Truelove S.C., Witts L.J. (1955). Cortisone in Ulcerative Colitis. Br. Med. J..

[120] Danese S., Fiorino G., Peyrin-Biroulet L. (2017). Filgotinib in Crohn’s Disease: JAK Is Back. Gastroenterology.

[121] Olivera P., Danese S., Peyrin-Biroulet L. (2017). Next generation of small molecules in inflammatory bowel disease. Gut.

[122] Lloyd-Price J., Arze C., Ananthakrishnan A.N., Schirmer M., Avila-Pacheco J., Poon T.W., Andrews E., Ajami N.J., Bonham K.S., Brislawn C.J. (2019). Multi-omics of the gut microbial ecosystem in inflammatory bowel diseases. Nature.

[123] Sandborn W.J., Su C., Sands B.E., D’Haens G.R., Vermeire S., Schreiber S., Danese S., Feagan B.G., Reinisch W., Niezychowski W. (2017). Tofacitinib as Induction and Maintenance Therapy for Ulcerative Colitis. N. Engl. J. Med..

[124] Fernandez-Clotet A., Castro-Poceiro J., Panes J. (2018). Tofacitinib for the treatment of ulcerative colitis. Expert Rev. Clin. Immunol..

[125] Lim W.C., Hanauer S.B. (2004). Controversies With Aminosalicylates in Inflammatory Bowel Disease. Rev. Gastroenterol. Disord..

[126] Rudrapatna V.A., Velayos F. (2019). Biosimilars for the Treatment of Inflammatory Bowel Disease. Pract. Gastroenterol..

[127] Rutgeerts P., Vermeire S., Van Assche G. (2009). Biological therapies for inflammatory bowel diseases. Gastroenterology.

[128] Colombel J.F., Sands B.E., Rutgeerts P., Sandborn W., Danese S., D’Haens G., Panaccione R., Loftus E.V., Sankoh S., Fox I. (2017). The safety of vedolizumab for ulcerative colitis and Crohn’s disease. Gut.

[129] Weisshof R., El Jurdi K., Zmeter N., Rubin D.T. (2018). Emerging Therapies for Inflammatory Bowel Disease. Adv. Ther..

[130] Streeter P.R., Berg E.L., Rouse B.T., Bargatze R.F., Butcher E.C. (1988). A tissue-specific endothelial cell molecule involved in lymphocyte homing. Nture.

[131] Sandborn W.J., Colombel J.F., Enns R., Feagan B.G., Hanauer S.B., Lawrance I.C., Panaccione R., Sanders M., Schreiber S., Targan S. (2005). Natalizumab induction and maintenance therapy for Crohn’s disease. N. Engl. J. Med..

[132] Danese S., Vuitton L., Peyrin-Biroulet L. (2015). Biologic agents for IBD: Practical insights. Nat. Rev. Gastroenterol. Hepatol..

[133] Yamamoto-Furusho J.K. (2018). Inflammatory bowel disease therapy: Blockade of cytokines and cytokine signaling pathways. Curr. Opin. Gastroenterol..

[134] Kuhn K.A., Manieri N.A., Liu T.C., Stappenbeck T.S. (2014). IL-6 stimulates intestinal epithelial proliferation and repair after injury. PLoS ONE.

[135] Bersudsky M., Luski L., Fishman D., White R.M., Ziv-Sokolovskaya N., Dotan S., Rider P., Kaplanov I., Aychek T., Dinarello C.A. (2014). Non-redundant properties of IL-1alpha and IL-1beta during acute colon inflammation in mice. Gut.

[136] Friedrich M., Pohin M., Powrie F. (2019). Cytokine Networks in the Pathophysiology of Inflammatory Bowel Disease. Immunity.

[137] Baert F., Noman M., Vermeire S., Van Assche G., D’Haens G., Carbonez A., Rutgeerts P. (2003). Influence of Immunogenicity on the Long-Term Efficacy of Infliximab in Crohn’s Disease. N. Engl. J. Med..

[138] Allez M., Karmiris K., Louis E., Van Assche G., Ben-Horin S., Klein A., Van der Woude J., Baert F., Eliakim R., Katsanos K. (2010). Report of the ECCO pathogenesis workshop on anti-TNF therapy failures in inflammatory bowel diseases: Definitions, frequency and pharmacological aspects. J. Crohn’s Colitis.

[139] Kim J.W., Lee C.K., Lee J.K., Jeong S.J., Oh S.J., Moon J.R., Kim H.S., Kim H.J. (2019). Long-term evolution of direct healthcare costs for inflammatory bowel diseases: A population-based study (2006–2015). Scand. J. Gastroenterol..

[140] Mak J.W., Sung J.J. (2019). The Use of Biologics and Biosimilar in Asian patients with IBD: Are we ready?. J. Gastroenterol. Hepatol..

[141] Dubinsky M. (2004). Azathioprine, 6-Mercaptopurine in Inflammatory Bowel Disease: Pharmacology, Efficacy, and Safety. Clin. Gastroenterol. Hepatol..

[142] Chandel S., Prakash A., Medhi B. (2015). Current scenario in inflammatory bowel disease: Drug development prospects. Pharmacol. Rep..

[143] Iacucci M., de Silva S., Ghosh S. (2010). Mesalazine in inflammatory bowel disease: A trendy topic once again?. Can. J. Gastroenterol..

[144] Carter M.J., Lobo A.J., Travis S.P. (2004). Guidelines for the management of inflammatory bowel disease in adults. Gut.

[145] Bergman R., Parkes M. (2006). Systematic review: The use of mesalazine in inflammatory bowel disease. Aliment. Pharmacol. Ther..

[146] D’Amico F., Parigi T.L., Fiorino G., Peyrin-Biroulet L., Danese S. (2019). Tofacitinib in the treatment of ulcerative colitis: Efficacy and safety from clinical trials to real-world experience. Therap. Adv. Gastroenterol..

[147] Campieri M., Ferguson A., Doe W., Persson T., Nilsson L.G. (1997). Oral budesonide is as eVective as oral prednisolone in active Crohn’s disease. Gut.

[148] Abdalla M.I., Herfarth H. (2016). Budesonide for the treatment of ulcerative colitis. Expert Opin. Pharmacother..

[149] Kuenzig M.E., Rezaie A., Kaplan G.G., Otley A.R., Steinhart A.H., Griffiths A.M., Benchimol E.I., Seow C.H. (2018). Budesonide for the Induction and Maintenance of Remission in Crohn’s Disease: Systematic Review and Meta-Analysis for the Cochrane Collaboration. J. Can. Assoc. Gastroenterol..

[150] Lichtenstein G.R., Feagan B.G., Cohen R.D., Salzberg B.A., Safdi M., Popp J.W., Langholff W., Sandborn W.J. (2018). Infliximab for Crohn’s Disease: More Than 13 Years of Real-world Experience. Inflamm. Bowel Dis..

[151] Papamichael K., Lin S., Moore M., Papaioannou G., Sattler L., Cheifetz A.S. (2019). Infliximab in inflammatory bowel disease. Ther. Adv. Chronic Dis..

[152] Melsheimer R., Geldhof A., Apaolaza I., Schaible T. (2019). Remicade((R)) (infliximab): 20 years of contributions to science and medicine. Biologics.

[153] Guidi L., Pugliese D., Armuzzi A. (2011). Update on the management of inflammatory bowel disease: Specific role of adalimumab. Clin. Exp. Gastroenterol..

[154] Tursi A., Elisei W., Faggiani R., Allegretta L., Valle N.D., Forti G., Franceschi M., Ferronato A., Gallina S., Larussa T. (2018). Effectiveness and safety of adalimumab to treat outpatient ulcerative colitis: A real-life multicenter, observational study in primary inflammatory bowel disease centers. Medicine (Baltimore).

[155] Armuzzi A., Felice C. (2013). Natalizumab in Crohn’s disease: Past and future areas of applicability. Ann. Gastroenterol..

[156] Caprilli D.G.R. (2008). Natalizumab in the treatment of Crohn’s disease. Biol. Targets Ther..

[157] Sandborn W.J., Abreu M.T., D’Haens G., Colombel J.F., Vermeire S., Mitchev K., Jamoul C., Fedorak R.N., Spehlmann M.E., Wolf D.C. (2010). Certolizumab pegol in patients with moderate to severe Crohn’s disease and secondary failure to infliximab. Clin. Gastroenterol. Hepatol..

[158] Schreiber S. (2011). Certolizumab pegol for the treatment of Crohn’s disease. Therap. Adv. Gastroenterol..

[159] Pelechas E., Voulgari P.V., Drosos A.A. (2019). Golimumab for Rheumatoid Arthritis. J. Clin. Med..

[160] Flamant M., Paul S., Roblin X. (2017). Golimumab for the treatment of ulcerative colitis. Expert Opin. Biol. Ther..

[161] Russi L., Scharl M., Rogler G., Biedermann L. (2017). The Efficacy and Safety of Golimumab as Third- or Fourth-Line Anti-TNF Therapy in Patients with Refractory Crohn’s Disease: A Case Series. Inflamm. Intest. Dis..

[162] Scribano M.L. (2018). Vedolizumab for inflammatory bowel disease: From randomized controlled trials to real-life evidence. World J. Gastroenterol..

[163] Weaver K.N., Gregory M., Syal G., Hoversten P., Hicks S.B., Patel D., Christophi G., Beniwal-Patel P., Isaacs K.L., Raffals L. (2019). Ustekinumab Is Effective for the Treatment of Crohn’s Disease of the Pouch in a Multicenter Cohort. Inflamm. Bowel Dis..

[164] Kotze P.G., Ma C., Almutairdi A., Panaccione R. (2018). Clinical utility of ustekinumab in Crohn’s disease. J. Inflamm. Res..

[165] Neurath M.F. (2014). Cytokines in inflammatory bowel disease. Nat. Rev. Immunol..

[166] Rutgeerts P.J., Fedorak R.N., Hommes D.W., Sturm A., Baumgart D.C., Bressler B., Schreiber S., Mansfield J.C., Williams M., Tang M. (2013). A randomised phase I study of etrolizumab (rhuMAb beta7) in moderate to severe ulcerative colitis. Gut.

[167] Vermeire S., Ghosh S., Panes J., Dahlerup J.F., Luegering A., Sirotiakova J., Strauch U., Burgess G., Spanton J., Martin S.W. (2011). The mucosal addressin cell adhesion molecule antibody PF- 00547,659 in ulcerative colitis: A randomised study. Gut.

[168] Feagan B.G., Sandborn W.J., Gasink C., Jacobstein D., Lang Y., Friedman J.R., Blank M.A., Johanns J., Gao L.L., Miao Y. (2016). Ustekinumab as induction and maintenance therapy for Crohn’s disease. N. Engl. J. Med..

[169] Savarino E., Bertani L., Ceccarelli L., Bodini G., Zingone F., Buda A., Facchin S., Lorenzon G., Marchi S., Marabotto E. (2019). Antimicrobial treatment with the fixed-dose antibiotic combination RHB-104 for Mycobacterium avium subspecies paratuberculosis in Crohn’s disease: Pharmacological and clinical implications. Expert Opin. Biol. Ther..

[170] Hanai H., Takeda Y., Eberhardson M., Gruber R., Saniabadi A.R., Winqvist O., Lofberg R. (2011). The mode of actions of the Adacolumn therapeutic leucocytapheresis in patients with inflammatory bowel disease: A concise review. Clin. Exp. Immunol..

[171] Hanai H., Watanabe F., Takeuchi K., Iida T., Yamada M., Iwaoka Y., Saniabadi A., Matsushita I., Sato Y., Tozawa K. (2003). Leukocyte adsorptive apheresis for the treatment of active ulcerative colitis: A prospective, uncontrolled, pilot study. Clin. Gastroenterol. Hepatol..

[172] Shimoyama T., Sawada K., Hiwatashi N., Sawada T., Matsueda K., Munakata A., Asakura H., Tanaka T., Kasukawa R., Kimura K. (2001). Safety and efficacy of granulocyte and monocyte adsorption apheresis in patients with active ulcerative colitis: A multicenter study. J. Clin. Apher..

[173] Dignass A.U., Eriksson A., Kilander A., Pukitis A., Rhodes J.M., Vavricka S. (2010). Clinical trial: Five or ten cycles of granulocyte-monocyte apheresis show equivalent efficacy and safety in ulcerative colitis. Aliment. Pharmacol. Ther..

[174] Bemelman W.A., S-ECCO collaborators (2018). Evolving Role of IBD Surgery. J. Crohn’s Colitis.

[175] Kuhn F., Klar E. (2015). Surgical Principles in the Treatment of Ulcerative Colitis. Viszeralmedizin.

[176] Frolkis A.D., Dykeman J., Negron M.E., Debruyn J., Jette N., Fiest K.M., Frolkis T., Barkema H.W., Rioux K.P., Panaccione R. (2013). Risk of surgery for inflammatory bowel diseases has decreased over time: A systematic review and meta-analysis of population-based studies. Gastroenterology.

[177] Wong D.J., Roth E.M., Feuerstein J.D., Poylin V.Y. (2019). Surgery in the age of biologics. Gastroenterol. Rep..

[178] Seifarth C., Kreis M.E., Grone J. (2015). Indications and Specific Surgical Techniques in Crohn’s Disease. Viszeralmedizin.

[179] Cima R.R., Pemberton J.H. (2004). Early surgical intervention in ulcerative colitis. Gut.

[180] Shen B., Lashner B.A. (2008). Diagnosis and Treatment of Pouchitis. Gasteroenterol. Gastroenterol Hepatol..

[181] Martin S.T., Vogel J.D. (2013). Restorative procedures in colonic crohn disease. Clin. Colon Rectal Surg..

[182] Fumery M., Dulai P.S., Meirick P., Farrell A.M., Ramamoorthy S., Sandborn W.J., Singh S. (2017). Systematic review with meta-analysis: Recurrence of Crohn’s disease after total colectomy with permanent ileostomy. Aliment. Pharmacol. Ther..

[183] Bemelman W.A., Warusavitarne J., Sampietro G.M., Serclova Z., Zmora O., Luglio G., de Buck van Overstraeten A., Burke J.P., Buskens C.J., Colombo F. (2018). ECCO-ESCP Consensus on Surgery for Crohn’s Disease. J. Crohn’s Colitis.

[184] Ahmed Ali U., Keus F., Heikens J.T., Bemelman W.A., Berdah S.V., Gooszen H.G., van Laarhoven C.J. (2009). Open versus laparoscopic (assisted) ileo pouch anal anastomosis for ulcerative colitis and familial adenomatous polyposis. Cochrane Database Syst. Rev..

[185] Beyer-Berjot L., Maggiori L., Birnbaum D., Lefevre J.H., Berdah S., Panis Y. (2013). A total laparoscopic approach reduces the infertility rate after ileal pouch-anal anastomosis: A 2-center study. Ann. Surg..

[186] Oresland T., Bemelman W.A., Sampietro G.M., Spinelli A., Windsor A., Ferrante M., Marteau P., Zmora O., Kotze P.G., Espin-Basany E. (2015). European evidence based consensus on surgery for ulcerative colitis. J. Crohn’s Colitis.

[187] Hilsden R.J., Meddings J.B., Verhoef M.J. (1999). Complementary and alternative medicine use by patients with inflammatory bowel disease: An Internet survey. Can. J. Gastroenterol..

[188] Hilsden R.J., Scott C.M., Verhoef M.J. (1998). Complementary Medicine Use by Patients With Inflammatory Bowel Disease. Am. J. Gastroenterol..

[189] Langhorst J., Wulfert H., Lauche R., Klose P., Cramer H., Dobos G.J., Korzenik J. (2015). Systematic review of complementary and alternative medicine treatments in inflammatory bowel diseases. J. Crohn’s Colitis.

[190] Triantafyllidi A., Xanthos T., Papalois A., Triantafillidis J.K. (2015). Herbal and plant therapy in patients with inflammatory bowel disease. Ann. Gastroenterol..

[191] Langmead L., Feakins R.M., Goldthorpe S., Holt H., Tsironi E., De Silva A., Jewell D.P., Rampton D.S. (2004). Randomized, double-blind, placebo-controlled trial of oral aloe vera gel for active ulcerative colitis. Aliment. Pharmacol. Ther..

[192] Fernandez-Banares F., Hinojosa J., Sanchez-Lombrana J.L., Navarro E., Martınez-Salmerón J.F., Garcıa-Pugés A., Gonzalez-Huix F., Riera J., Gonzalez-Lara V., Domınguez-Abascal F. (1999). Randomized Clinical Trial of Plantago ovata Seeds (Dietary Fiber) as Compared With Mesalamine in Maintaining Remission in Ulcerative Colitis. Am. J. Gastroenterol..

[193] Sandborn W.J., Targan S.R., Byers V.S., Rutty D.A., Mu H., Zhang X., Tang T. (2013). Andrographis paniculata extract (HMPL-004) for active ulcerative colitis. Am. J. Gastroenterol..

[194] Zhu Q., Zheng P., Chen X., Zhou F., He Q., Yang Y. (2018). Andrographolide presents therapeutic effect on ulcerative colitis through the inhibition of IL-23/IL-17 axis. Am. J. Transl. Res..

[195] Sun J., Shen X., Dong J., Wang H., Zuo L., Zhao J., Zhu W., Li Y., Gong J., Li J. (2015). Tripterygium wilfordii Hook F as Maintenance Treatment for Crohn’s Disease. Am. J. Med. Sci..

[196] Kinnucan J. (2018). Use of Medical Cannabis in Patients With Inflammatory Bowel Disease. Gastroenterol. Hepatol..

[197] Little T.J., Shuker D.M., Colegrave N., Day T., Graham A.L. (2010). The coevolution of virulence: Tolerance in perspective. PLoS Pathog..

[198] Elliott D.E., Urban J.F., Argo C.K., Weinstock J.V. (2000). Does the failure to acquire helminthic parasites predispose to Crohn’s disease?. FASEB J..

[199] Summers R.W., Elliott D.E., Urban J.F., Thompson R., Weinstock J.V. (2005). Trichuris suis therapy in Crohn’s disease. Gut.

[200] Heylen M., Ruyssers N.E., Gielis E.M., Vanhomwegen E., Pelckmans P.A., Moreels T.G., De Man J.G., De Winter B.Y. (2014). Of worms, mice and man: An overview of experimental and clinical helminth-based therapy for inflammatory bowel disease. Pharmacol. Ther..

[201] Johnston M.J., MacDonald J.A., McKay D.M. (2009). Parasitic helminths: A pharmacopeia of anti-inflammatory molecules. Parasitology.

[202] Scholmerich J., Fellermann K., Seibold F.W., Rogler G., Langhorst J., Howaldt S., Novacek G., Petersen A.M., Bachmann O., Matthes H. (2017). A Randomised, Double-blind, Placebo-controlled Trial of Trichuris suis ova in Active Crohn’s Disease. J. Crohn’s Colitis.

[203] Burakoff R., Pabby V., Onyewadume L., Odze R., Adackapara C., Wang W., Friedman S., Hamilton M., Korzenik J., Levine J. (2015). Blood-based biomarkers used to predict disease activity in Crohn’s disease and ulcerative colitis. Inflamm. Bowel Dis..

[204] Dieckgraefe B.K., Stenson W.F., Korzenik J.R., Swanson P.E., Harrington C.A. (2000). Analysis of mucosal gene expression in inflammatory bowel disease by parallel oligonucleotide arrays. Physiol. Genom..

[205] Burczynski M.E., Peterson R.L., Twine N.C., Zuberek K.A., Brodeur B.J., Casciotti L., Maganti V., Reddy P.S., Strahs A., Immermann F. (2006). Molecular classification of Crohn’s disease and ulcerative colitis patients using transcriptional profiles in peripheral blood mononuclear cells. J. Mol. Diagn..

[206] Wu F., Guo N.J., Tian H., Marohn M., Gearhart S., Bayless T.M., Brant S.R., Kwon J.H. (2011). Peripheral blood microRNAs distinguish active ulcerative colitis and Crohn’s disease. Inflamm. Bowel Dis..

[207] Feagan B.G., Macdonald J.K. (2012). Oral 5-aminosalicylic acid for induction of remission in ulcerative colitis. Cochrane Database Syst. Rev..

[208] Porro G.B., Cassinotti A., Ferrara E., Maconi G., Ardizzone S. (2007). Review article: The management of steroid dependency in ulcerative colitis. Aliment. Pharmacol. Ther..

[209] Rufo P.A., Bousvaros A. (2006). Current Therapy of Inflammatory Bowel Disease in Children. Pediatr. Drugs.

[210] D’Haens G.R., Sartor R.B., Silverberg M.S., Petersson J., Rutgeerts P. (2014). Future directions in inflammatory bowel disease management. J. Crohn’s Colitis.

[211] Gasche C.S., Brynskov J., D’Haens J., Hanauer S.B., Irvine E.J., Jewell D.P., Rlachmilewitz D., Sachar D.B., Sandborn W.J. (2000). A Simple Classification of CI rohn’s Disease: Report of the Working -Party for the World-congresses of Gastroenterology, Vienna 1998. Inflamm. Bowel Dis..

[212] Silverberg M.S., Satsangi J., Ahmad T., Arnott I.D., Bernstein C.N., Brant S.R., Caprilli R., Colombel J.F., Gasche C., Geboes K. (2005). Toward an integrated clinical, molecular and serological classification of inflammatory bowel disease: Report of a Working Party of the 2005 Montreal World Congress of Gastroenterology. Can. J. Gastroenterol..

[213] Dassopoulos T., Nguyen G.C., Bitton A., Bromfield G.P., Schumm L.P., Wu Y., Elkadri A., Regueiro M., Siemanowski B., Torres E.A. (2007). Assessment of reliability and validity of IBD phenotyping within the National Institutes of Diabetes and Digestive and Kidney Diseases (NIDDK) IBD Genetics Consortium (IBDGC). Inflamm. Bowel Dis..

[214] Clish C.B. (2015). Metabolomics: An emerging but powerful tool for precision medicine. Cold Spring Harb. Mol. Case Stud..

[215] Johnston C., Dunn W., Broadhurst D., Brown M., Goodacre R., Campbell S., Makin A., Newman W., Watson A.J. (2010). T1259 Serum Metabolite Profiles Differentiate Crohn’s Disease From Ulcerative Colitis and From Healthy Controls. Gastroenterology.

[216] Kolho K.L., Pessia A., Jaakkola T., de Vos W.M., Velagapudi V. (2017). Faecal and Serum Metabolomics in Paediatric Inflammatory Bowel Disease. J. Crohn’s Colitis.

[217] Jansson J., Willing B., Lucio M., Fekete A., Dicksved J., Halfvarson J., Tysk C., Schmitt-Kopplin P. (2009). Metabolomics reveals metabolic biomarkers of Crohn’s disease. PLoS ONE.

[218] Bjerrum J.T., Nielsen O.H., Hao F., Tang H., Nicholson J.K., Wang Y., Olsen J. (2010). Metabonomics in Ulcerative Colitis: Diagnostics, Biomarker Identification, And Insight into the Pathophysiology. J. Proteome Res..

[219] Nash P.T., Florin T.H. (2005). Tumour necrosis factor inhibitors. J. Med. J. Austr..

[220] Hamlin P.J., Warren L., Everett S.M. (2011). Establishing a biologics service for patients with inflammatory bowel disease. Frontline Gastroenterol..

[221] Mulcahy A.W., Hlavka J.P., Case S.R. (2017). Biosimilar cost savings in the United States: Initial experience and future potential. Rand Health Q..

[222] Kochar B., Barnes E.L., Long M.D., Cushing K.C., Galanko J., Martin C.F., Raffals L.E., Sandler R.S. (2018). Depression Is Associated With More Aggressive Inflammatory Bowel Disease. Am. J. Gastroenterol..

[223] Crohn’s & Colitis Australia (2016). Final Report of the First Audit of the Organisation and Provision of IBD Services in Australia.

[224] Knowles S., Andrews J.M., Porter A. (2018). Predictors of Impaired Mental Health and Support Seeking in Adults with Inflammatory Bowel Disease: An Online Survey. Gastroenterol. Nurs..

[225] Massuger W., Moore G.T., Andrews J.M., Kilkenny M.F., Reyneke M., Knowles S., Purcell L., Alex G., Buckton S., Page A.T. (2019). The Crohn’s & Colitis Australia inflammatory bowel disease audit: Measuring the quality of care in Australia. Intern. Med. J..

[226] Nigro G., Angelini G., Grosso S.B., Caula G., Sategna-Guidetti C. (2001). Psychiatric Predictors of Noncompliance in Inflammatory Bowel Disease Psychiatry and Compliance. J. Clin. Gastroenterol..

[227] Tulp M., Bohlin L. (2002). Functional versus chemical diversity: Is biodiversity important for drug discovery?. Trends Pharmacol. Sci..

[228] Grabowski K., Baringhaus K.H., Schneider G. (2008). Scaffold diversity of natural products: Inspiration for combinatorial library design. Nat. Prod. Rep..

[229] Harvey A.L. (2008). Natural products in drug discovery. Drug Discov. Today.

[230] Wangchuk P., Loukas A., Mandal S., Mandal V., Konishi T. (2018). Techniques and Technologies for the Biodiscovery of Novel Small Molecule Drug Lead Compounds From Natural Products. Natural Products and Drug Discovery: An Integrated Approach.

[231] Macielag M.J., Dougherty T.J., Pucci M.J. (2012). Chemical Properties of Antimicrobials and Their Uniqueness. Antibiotic Discovery and Development.

[232] Wang K., Xiao J., Liu X., Jiang Z., Zhan Y., Yin T., He L., Zhang F., Xing S., Chen B. (2019). AICD: An integrated anti-inflammatory compounds database for drug discovery. Sci. Rep..

[233] Aswad M., Rayan M., Abu-Lafi S., Falah M., Raiyn J., Abdallah Z., Rayan A. (2018). Nature is the best source of anti-inflammatory drugs: Indexing natural products for their anti-inflammatory bioactivity. Inflamm. Res..

[234] Shiomi Y., Nishiumi S., Ooi M., Hatano N., Shinohara M., Yoshie T., Kondo Y., Furumatsu K., Shiomi H., Kutsumi H. (2011). GCMS-based metabolomic study in mice with colitis induced by dextran sulfate sodium. Inflamm. Bowel Dis..

[235] Hisamatsu T., Okamoto S., Hashimoto M., Muramatsu T., Andou A., Uo M., Kitazume M.T., Matsuoka K., Yajima T., Inoue N. (2012). Novel, objective, multivariate biomarkers composed of plasma amino acid profiles for the diagnosis and assessment of inflammatory bowel disease. PLoS ONE.

[236] Goyal N., Rana A., Ahlawat A., Bijjem K.R., Kumar P. (2014). Animal models of inflammatory bowel disease: A review. Inflammopharmacology.

[237] Mizoguchi A. (2012). Animal models of inflammatory bowel disease. Prog. Mol. Biol. Transl. Sci..

[238] Kiesler P., Fuss I.J., Strober W. (2015). Experimental Models of Inflammatory Bowel Diseases. Cell Mol. Gastroenterol. Hepatol..

[239] Morgan R.E., Ahn S., Nzimiro S., Fotie J., Phelps M.A., Cotrill J., Yakovich A.J., Sackett D.L., Dalton J.T., Werbovetz K.A. (2008). Inhibitors of tubulin assembly identified through screening a compound library. Chem. Biol. Drug Des..

[240] Ahmad T.B., Liu L., Kotiw M., Benkendorff K. (2018). Review of anti-inflammatory, immune-modulatory and wound healing properties of molluscs. J. Ethnopharmacol..

[241] Azab A., Nassar A., Azab A.N. (2016). Anti-Inflammatory Activity of Natural Products. Molecules.

[242] Peng J., Zheng T.T., Li X., Liang Y., Wang L.J., Huang Y.C., Xiao H.T. (2019). Plant-Derived Alkaloids: The Promising Disease-Modifying Agents for Inflammatory Bowel Disease. Front. Pharmacol..

[243] Salaritabar A., Darvishi B., Hadjiakhoondi F., Manayi A., Sureda A., Nabavi S.F., Fitzpatrick L.R., Nabavi S.M., Bishayee A. (2017). Therapeutic potential of flavonoids in inflammatory bowel disease: A comprehensive review. World J. Gastroenterol..

[244] Leong D.J., Choudhury M., Hanstein R., Hirsh D.M., Kim S.J., Majeska R.J., Schaffler M.B., Hardin J.A., Spray D.C., Goldring M.B. (2014). Green tea polyphenol treatment is chondroprotective, anti-inflammatory and palliative in a mouse post-traumatic osteoarthritis model. Arthritis Res. Ther..

[245] Takahara M., Takaki A., Hiraoka S., Adachi T., Shimomura Y., Matsushita H., Nguyen T.T.T., Koike K., Ikeda A., Takashima S. (2019). Berberine improved experimental chronic colitis by regulating interferon-gamma- and IL-17A-producing lamina propria CD4(+) T cells through AMPK activation. Sci. Rep..

[246] Yu X.T., Xu Y.F., Huang Y.F., Qu C., Xu L.Q., Su Z.R., Zeng H.F., Zheng L., Yi T.G., Li H.L. (2018). Berberrubine attenuates mucosal lesions and inflammation in dextran sodium sulfate-induced colitis in mice. PLoS ONE.

[247] Wang G., Hu Z., Song X., Cui Q., Fu Q., Jia R., Zou Y., Li L., Yin Z. (2017). Analgesic and Anti-Inflammatory Activities of Resveratrol through Classic Models in Mice and Rats. Evid. Based Complement. Alternat. Med..

[248] Wangchuk P., Navarro S., Shepherd C., Keller P.A., Pyne S.G., Loukas A. (2015). Diterpenoid alkaloids of Aconitum laciniatum and mitigation of inflammation by 14-O-acetylneoline in a murine model of ulcerative colitis. Sci. Rep..

[249] Verma N., Meena N.K., Majumdar I., Paul J. (2017). Role of Bromelain as Herbal Anti-Inflammatory Compound Using In Vitro and In Vivo Model of Colitis. J. Autoimmune Dis..

[250] Shepherd C., Giacomin P., Navarro S., Miller C., Loukas A., Wangchuk P. (2018). A medicinal plant compound, capnoidine, prevents the onset of inflammation in a mouse model of colitis. J. Ethnopharmacol..

[251] Cho W., Nam J.W., Kang H.J., Windono T., Seo E.K., Lee K.T. (2009). Zedoarondiol isolated from the rhizoma of Curcuma heyneana is involved in the inhibition of iNOS, COX-2 and pro-inflammatory cytokines via the downregulation of NF-kappaB pathway in LPS-stimulated murine macrophages. Int. Immunopharmacol..

[252] Chaninani-Wu N. (2003). Safety and Anti-Inflammatory Activity of Curcumin: A Component of Tumeric (*Curcuma longa*). J. Altern. Complement. Med..

[253] Zou W., Xiao Z., Wen X., Luo J., Chen S., Cheng Z., Xiang D., Hu J., He J. (2016). The anti-inflammatory effect of Andrographis paniculata (Burm. f.) Nees on pelvic inflammatory disease in rats through down-regulation of the NF-kappaB pathway. BMC Complement. Altern. Med..

[254] Rodriguez-Canales M., Jimenez-Rivas R., Canales-Martinez M.M., Garcia-Lopez A.J., Rivera-Yanez N., Nieto-Yanez O., Ledesma-Soto Y., Sanchez-Torres L.E., Rodriguez-Sosa M., Terrazas L.I. (2016). Protective Effect of Amphipterygium adstringens Extract on Dextran Sulphate Sodium-Induced Ulcerative Colitis in Mice. Mediat. Inflamm..

[255] Choi R.J., Shin E.M., Jung H.A., Choi J.S., Kim Y.S. (2011). Inhibitory effects of kaurenoic acid from Aralia continentalis on LPS-induced inflammatory response in RAW264.7 macrophages. Phytomedicine.

[256] Bhanu P.K., Kotakadi V.S. (2014). Anti-inflammatory Effect of Basella rubra on Oxazolone-induced Colitis in Rat. Am. J. Phytomed. Clin. Ther..

[257] Schink A., Naumoska K., Kitanovski Z., Kampf C.J., Frohlich-Nowoisky J., Thines E., Poschl U., Schuppan D., Lucas K. (2018). Anti-inflammatory effects of cinnamon extract and identification of active compounds influencing the TLR2 and TLR4 signaling pathways. Food Funct..

[258] Castro J.P., Ocampo Y.C., Franco L.A. (2014). In vivo and in vitro anti-inflammatory activity of Cryptostegia grandiflora Roxb. ex R. Br. leaves. Biol. Res..

[259] Dutra R.C., Claudino R.F., Bento A.F., Marcon R., Schmidt E.C., Bouzon Z.L., Pianowski L.F., Calixto J.B. (2011). Preventive and therapeutic euphol treatment attenuates experimental colitis in mice. PLoS ONE.

[260] Noh E.J., Ahn K.S., Shin E.M., Jung S.H., Kim Y.S. (2006). Inhibition of lipopolysaccharide-induced iNOS and COX-2 expression by dehydroevodiamine through suppression of NF-kappaB activation in RAW 264.7 macrophages. Life Sci..

[261] Yang Z., Lu W., Ma X., Song D. (2012). Bioassay-guided isolation of an alkaloid with antiangiogenic and antitumor activities from the extract of Fissistigma cavaleriei root. Phytomedicine.

[262] Barbieri A., Quagliariello V., Del Vecchio V., Falco M., Luciano A., Amruthraj N.J., Nasti G., Ottaiano A., Berretta M., Iaffaioli R.V. (2017). Anticancer and Anti-Inflammatory Properties of Ganoderma lucidum Extract Effects on Melanoma and Triple-Negative Breast Cancer Treatment. Nutrients.

[263] da Silva V.C., de Araujo A.A., de Souza Araujo D.F., Souza Lima M.C.J., Vasconcelos R.C., de Araujo Junior R.F., Langasnner S.M.Z., de Freitas Fernandes Pedrosa M., de Medeiros C., Guerra G.C.B. (2018). Intestinal Anti-Inflammatory Activity of the Aqueous Extract from Ipomoea asarifolia in DNBS-Induced Colitis in Rats. Int. J. Mol. Sci..

[264] Huo X., Zhang L., Gao L., Guo Y., Zhang L., Li L., Si J., Cao L. (2015). Antiinflammatory and Analgesic Activities of Ethanol Extract and Isolated Compounds from Millettia pulchra. Biol. Pharm. Bull..

[265] Burgess K., Rankin N., Weidt S., Padmanabhan S. (2014). Metabolomics. Handbook of Pharmacogenomics and Stratified Medicine.

[266] Wang C.Z., Yu C., Wen X.D., Chen L., Zhang C.F., Calway T., Qiu Y., Wang Y., Zhang Z., Anderson S. (2016). American Ginseng Attenuates Colitis-Associated Colon Carcinogenesis in Mice: Impact on Gut Microbiota and Metabolomics. Cancer Prev. Res. (Phila).

[267] Safarpour A.R., Kaviyani F., Sepehrimanesh M., Ahmadi N., Hosseinabadi O.K., Tanideh N., Showraki N. (2015). Antioxidant and Anti-Inflammatory Effects of Gel and Aqueous Extract of Melilotus officinalis L. in Induced Ulcerative Colitis: A Rattus norvegicus Model. Ann. Colorectal Res..

[268] Khodayar B., Farzaei M.H., Abdolghaffari A.H., Bahramsoltani R., Baeeri M., Sabbagh Ziarani F., Mohammadi M., Rahimi R., Abdollahi M. (2018). The Protective Effect of the Gallic Acid Against TNBS-induced Ulcerative Colitis in Rats: Role of Inflammatory Parameters. JIMC.

[269] Toledo T.R., Dejani N.N., Monnazzi L.G., Kossuga M.H., Berlinck R.G., Sette L.D., Medeiros A.I. (2014). Potent anti-inflammatory activity of pyrenocine A isolated from the marine-derived fungus Penicillium paxilli Ma(G)K. Mediat. Inflamm..

[270] Amri O., Zekhnini A., Bouhaimi A., Tahrouch S., Hatimi A. (2017). Anti-inflammatory Activity of Methanolic Extract from Pistacia atlantica Desf. Leaves. Pharmacogn. J..

[271] Algieri F., Rodriguez-Nogales A., Garrido-Mesa N., Zorrilla P., Burkard N., Pischel I., Sievers H., Benedek B., Feistel B., Walbroel B. (2014). Intestinal anti-inflammatory activity of the Serpylli herba extract in experimental models of rodent colitis. J. Crohn’s Colitis.

[272] Kang H.S., Kim Y.H., Lee C.S., Lee J.J., Choi I., Pyun K.H. (1996). Anti-inflammatory effects of Stephania tetrandra S. Moore on interleukin-6 production and experimental inflammatory disease models. Mediat. Inflamm..

[273] Sutradhar R.K., Rahman A.M., Ahmad M., Bachar S.C., Saha A., Guha S.K. (2006). Bioactive Alkaloid from Sida cordifolia Linn. wit Analgesic and Anti-Inflammatory Activities. IJPT.

[274] Yun K.J., Min B.S., Kim J.Y., Lee K.T. (2007). Styraxoside A Isolated from the Stem Bark of Styrax japonica Inhibits Lipopolysaccharide-Induced Expression of Inducible Nitric Oxide Synthase and Cyclooxygenase-2 in RAW 264.7 Cells by Suppressing Nuclear Factor-kappa B Activation. Biol. Pharm. Bull..

[275] Xu J., Yi M., Ding L., He S. (2019). A Review of Anti-Inflammatory Compounds from Marine Fungi, 2000–2018. Mar. Drugs.

[276] Gu B.B., Jiao F.R., Wu W., Jiao W.H., Li L., Sun F., Wang S.P., Yang F., Lin H.W. (2018). Preussins with Inhibition of IL-6 Expression from Aspergillus flocculosus 16D-1, a Fungus Isolated from the Marine Sponge Phakellia fusca. J. Nat. Prod..

[277] Niu S., Xie C.L., Xia J.M., Luo Z.H., Shao Z., Yang X.W. (2018). New anti-inflammatory guaianes from the Atlantic hydrotherm-derived fungus Graphostroma sp. MCCC 3A00421. Sci. Rep..

[278] Niu S., Xie C.-L., Zhong T., Xu W., Luo Z.-H., Shao Z., Yang X.-W. (2017). Sesquiterpenes from a deep-sea-derived fungus Graphostroma sp. MCCC 3A00421. Tetrahedron.

[279] Renner M.K., Jensen P.R., Fenical W. (2000). Mangicols: Structures and Biosynthesis of A New Class of Sesterterpene Polyols from a Marine Fungus of the Genus Fusarium. J. Org. Chem..

[280] Yang X., Kang M.C., Li Y., Kim E.A., Kang S.M., Jeon Y.J. (2017). Asperflavin, an Anti-Inflammatory Compound Produced by a Marine-Derived Fungus, Eurotium amstelodami. Molecules.

[281] Patel G., Patil M.D., Soni S., Khobragade T.P., Chisti Y., Banerjee U.C. (2016). Production of mycophenolic acid by Penicillium brevicompactum-A comparison of two methods of optimization. Biotechnol. Rep. (Amst).

[282] Khan N.T. (2017). Cyclosporin A Production from Tolipocladium inflatum. Gen. Med..

[283] Mehling M., Johnson T.A., Antel J., Kappos L., Bar-Or A. (2011). Clinical immunology of the sphingosine 1-phosphate receptor modulator fingolimod (FTY720) in multiple sclerosis. Neurology.

[284] Elsayed E.A., El Enshasy H., Wadaan M.A., Aziz R. (2014). Mushrooms: A potential natural source of anti-inflammatory compounds for medical applications. Mediat. Inflamm..

[285] Deshmukh S.K., Verekar S.A., Periyasamy G., Ganguli B.N., Satyanarayana T., Johri B.N., Prakash A. (2012). Fungi: A Potential Source of Anti-inflammatory Compounds. Microorganisms in Sustainable Agriculture and Biotechnology.

[286] Maizels R.M., Smits H.H., McSorley H.J. (2018). Modulation of Host Immunity by Helminths: The Expanding Repertoire of Parasite Effector Molecules. Immunity.

[287] Kahl J., Brattig N., Liebau E. (2018). The Untapped Pharmacopeic Potential of Helminths. Trends Parasitol..

[288] Melon A., Wang A., Phan V., McKay D.M. (2010). Infection with Hymenolepis diminuta is more effective than daily corticosteroids in blocking chemically induced colitis in mice. J. Biomed. Biotechnol..

[289] Khan W.I., Blennerhasset P.A., Varghese A.K., Chowdhury S.K., Omsted P., Deng Y., Collins S.M. (2002). Intestinal nematode infection ameliorates experimental colitis in mice. Infect. Immun..

[290] Heylen M., Ruyssers N.E., Nullens S., Schramm G., Pelckmans P.A., Moreels T.G., De Man J.G., De Winter B.Y. (2015). Treatment with egg antigens of Schistosoma mansoni ameliorates experimental colitis in mice through a colonic T-cell-dependent mechanism. Inflamm. Bowel Dis..

[291] Wangchuk P., Shepherd C., Constantinoiu C., Ryan R.Y.M., Kouremenos K.A., Becker L., Jones L., Buitrago G., Giacomin P., Wilson D. (2019). Hookworm-derived metabolites suppress pathology in a mouse model of colitis and inhibit secretion of key inflammatory cytokines in primary human leukocytes. Infect. Immun..

[292] Wangchuk P., Kouremenos K., Eichenberger R.M., Pearson M., Susianto A., Wishart D.S., McConville M.J., Loukas A. (2019). Metabolomic profiling of the excretory-secretory products of hookworm and whipworm. Metabolomics.

[293] Wangchuk P., Constantinoiu C., Eichenberger R.M., Field M., Loukas A. (2019). Characterization of tapeworm metabolites and their reported biological activities. Molecules.

[294] Bettenworth D., Buyse M., Bohm M., Mennigen R., Czorniak I., Kannengiesser K., Brzoska T., Luger T.A., Kucharzik T., Domschke W. (2011). The tripeptide KdPT protects from intestinal inflammation and maintains intestinal barrier function. Am. J. Pathol..

[295] Dalmasso G., Charrier–Hisamuddin L., Nguyen H.T., Yan Y., Sitaraman S., Merlin D. (2008). PepT1-Mediated Tripeptide KPV Uptake Reduces Intestinal Inflammation. Gastroenterology.

[296] Kovacs-Nolan J., Zhang H., Ibuki M., Nakamori T., Yoshiura K., Turner P.V., Matsui T., Mine Y. (2012). The PepT1-transportable soy tripeptide VPY reduces intestinal inflammation. Biochim. Biophys. Acta.

[297] Wada S., Sato K., Ohta R., Wada E., Bou Y., Fujiwara M., Kiyono T., Park E.Y., Aoi W., Takagi T. (2013). Ingestion of low dose pyroglutamyl leucine improves dextran sulfate sodium-induced colitis and intestinal microbiota in mice. J. Agric. Food Chem..

[298] Caceres C.C., Bansal P.S., Navarro S., Wilson D., Don L., Giacomin P., Loukas A., Daly N.L. (2017). An engineered cyclic peptide alleviates symptoms of inflammation in a murine model of inflammatory bowel disease. J. Biol. Chem..

[299] Cobos C., Bansal P.S., Jones L., Wangchuk P., Wilson D., Loukas A., Daly N.L. (2018). Engineering of an Anti-Inflammatory Peptide Based on the Disulfide-Rich Linaclotide Scaffold. Biomedicines.

